# From a microscopic inertial active matter model to the Schrödinger equation

**DOI:** 10.1038/s41467-022-35635-1

**Published:** 2023-03-09

**Authors:** Michael te Vrugt, Tobias Frohoff-Hülsmann, Eyal Heifetz, Uwe Thiele, Raphael Wittkowski

**Affiliations:** 1 Institut für Theoretische Physik, Westfälische Wilhelms-Universität Münster, 48149 Münster, Germany; 2 Center for Soft Nanoscience (SoN), Westfälische Wilhelms-Universität Münster, 48149 Münster, Germany; 3 Porter School of the Environment and Earth Sciences, Tel Aviv University, 69978 Tel Aviv, Israel; 4 Center for Nonlinear Science (CeNoS), Westfälische Wilhelms-Universität Münster, 48149 Münster, Germany; 5 Center for Multiscale Theory and Computation (CMTC), Westfälische Wilhelms-Universität Münster, 48149 Münster, Germany

**Keywords:** Statistical physics, Nonlinear phenomena, Quantum mechanics, Fluid dynamics, Dark energy and dark matter

## Abstract

Active field theories, such as the paradigmatic model known as ‘active model B+’, are simple yet very powerful tools for describing phenomena such as motility-induced phase separation. No comparable theory has been derived yet for the underdamped case. In this work, we introduce active model I+, an extension of active model B+ to particles with inertia. The governing equations of active model I+ are systematically derived from the microscopic Langevin equations. We show that, for underdamped active particles, thermodynamic and mechanical definitions of the velocity field no longer coincide and that the density-dependent swimming speed plays the role of an effective viscosity. Moreover, active model I+ contains an analog of the Schrödinger equation in Madelung form as a limiting case, allowing one to find analoga of the quantum-mechanical tunnel effect and of fuzzy dark matter in active fluids. We investigate the active tunnel effect analytically and via numerical continuation.

## Abstract (web summary)

Active field theories are powerful tools to explain phenomena such as motility-induced phase separation. The authors report an active analogue to the quantum mechanics tunneling effect, showing similarity to the Schrödinger equation, by introducing an extended model applicable to active particles with inertia.

## Introduction

The study of active particles has become one of the fastest-growing fields of research in soft matter physics and statistical mechanics due to the enormous number of interesting effects that active matter can exhibit. Among these effects are a plethora of analogies between active matter and quantum mechanics. This includes Bose-Einstein condensation^1–3^, Fermi-Dirac statistics applied to polymer loops^4^, Hall viscosities^5,6^, orientational order in systems of fully symmetric particles^7,8^, Schrödinger-type dynamics in polar liquids^9^, spin-orbit coupling^10^, stationary Schrödinger equations for velocity distributions^11^, time crystals^12^, and topological effects^13^. A very simple yet extremely powerful description for active matter is given by scalar active field theories such as active model B (AMB)^14^ and the more general active model B+ (AMB+)^15^. These provide a minimal description for effects such as active phase separation and have led to crucial insights into the thermodynamics of active matter^16–20^.

While such field theories have also been coupled to the momentum-conserving dynamics of the solvent^18,19,21,22^, the inertia of the active particles themselves has been ignored in this context. However, recent experiments^23–25^ have found that the inertia of active particles is important in a variety of contexts. Moreover, theoretical and experimental studies have found a number of unusual effects associated with inertial active matter^26^, ranging from self-sustained temperature gradients^27^ through restored equilibrium crystallization^28^ to damping-dependent phase boundaries^29^. Consequently, there has been a strongly increasing recent interest in inertial active matter^30–34^.

Field theories for inertial active matter have been derived in previous work^29,35,36^ as extensions of the active phase field crystal (PFC) model^37–40^. Active PFC models can be derived as an approximation of dynamical density functional theory (DDFT)^41^, and have two disadvantages compared to AMB+. First, they rely on the close-to-equilibrium (adiabatic) approximation that DDFT is based on, and second, they require two order parameter fields (density *ρ* and polarization **P**) rather than just one, making them more complex. In contrast to PFC models, to the best of our knowledge, no extension of AMB+ to the underdamped case has been derived yet. A second gap is that up to now formal analogies between quantum mechanics and active matter, which are known to be useful in other contexts^4,11^, have not been exploited for the collective dynamics of inertial active matter.

In this work, we close both of these gaps. The first main result is an extension of AMB+ to particles with inertia that we refer to as active model I+. It is found that thermodynamic and mechanical definitions of the velocity field lead to different results in the active case, and that the density-dependent swimming speed of active particles gives rise to an effective viscosity of the active fluid. As a second main result, we show that active model I (the underdamped analogon of AMB) contains, as a limiting case, hydrodynamic equations that are formally equivalent to the Madelung equations^42,43^, which constitute a hydrodynamic representation of the Schrödinger equation^44,45^. This allows us to find analoga of fuzzy dark matter and the quantum-mechanical tunnel effect in an active fluid. A numerical investigation of the active tunnel effect using continuation methods shows that it also occurs when the approximations required for the active-quantum mapping are not exactly satisfied. This implies its robustness as physical phenomenon. In the Methods, we present a microscopic derivation of active model I+ using the well-established interaction-expansion method^46–50^.

## Results

### Active model I+

In this section, we introduce active model I+.

Our starting point is AMB+^15^, which is given by

1 $$\dot{\rho }={{{{{{{{{\boldsymbol{\nabla }}}}}}}}}}\cdot \,\left(M\rho ({{{{{{{{{\boldsymbol{\nabla }}}}}}}}}}({\;f}_{{{{{{{{{{\rm{o}}}}}}}}}}}^{{\prime} }(\rho )-\kappa {{{{{{{{{{\boldsymbol{\nabla }}}}}}}}}}}^{2}\rho+\lambda {({{{{{{{{{\boldsymbol{\nabla }}}}}}}}}}\rho )}^{2})-\xi ({{{{{{{{{{\boldsymbol{\nabla }}}}}}}}}}}^{2}\rho ){{{{{{{{{\boldsymbol{\nabla }}}}}}}}}}\rho )\right)$$

with a local particle number density *ρ*(**r**, *t*) depending on position **r** and time *t*, a mobility parameter *M*, an (overdamped) free-energy density *f*_o_(*ρ*) typically assumed to be a fourth-order polynomial, the notation ${f}_{{{{{{{{{{\rm{o}}}}}}}}}}}^{{\prime} }={\partial }_{\rho }\,{f}_{{{{{{{{{{\rm{o}}}}}}}}}}}$, and constants *κ*, *λ*, and *ξ*. An overdot denotes a partial derivative with respect to *t*. The model (1) is overdamped. Typically, one introduces AMB+ with a constant mobility *M*_0_ rather than with a density-dependent mobility *Mρ* as done here. The assumption of a constant mobility is valid only for uniform states or close to a critical point, but qualitatively reasonable also in other cases. The purpose of this approximation is to get a noise that is additive rather than multiplicative^20^. Here, we do not have a noise term since our microscopic derivation interprets *ρ* as an ensemble-averaged density^51^. By setting *ξ* = 0 in Eq. (1), one obtains AMB^14^. AMB, in turn, can be thought of as a minimal extension of the Cahn-Hilliard equation^52^ to the active case. The name “active model B” is based on the classification of Hohenberg and Halperin^53^, where AMB is a model of type B (conserved scalar order parameter).

An important feature of AMB and AMB+, which distinguishes them from passive field theories, is that the right-hand side of Eq. (1) cannot be written as a gradient dynamics, i.e., in terms of the functional derivative of a free energy^14,54^. In addition, AMB+ allows (unlike AMB) for circulating currents in steady state^15^. One can derive AMB+ either phenomenologically by writing down a general theory of a certain order in gradients and fields (top-down approach) or microscopically by explicit coarse-graining of the microscopic equations of motion of active particles (bottom-up approach)^20^. Here, the bottom-up approach has the advantage of providing explicit expressions for the coefficients appearing in the model (predictive theory)^48,49^ and giving a clearer insight into the origin of the various terms and the approximations required to get them.

Since AMB+ is overdamped, it does not take the inertia of the active particles into account. In this work, we obtain via a microscopic derivation an extension of AMB+ to the underdamped case, which we will refer to as active model I+ (AMI+), with “I” standing for “inertial”. It is given by

2 $$\dot{\rho }=-{{{{{{{{{\boldsymbol{\nabla }}}}}}}}}}\cdot (\rho {{{{{{{{{\bf{v}}}}}}}}}})+\frac{1}{2{D}_{{{{{{{{{{\rm{R}}}}}}}}}}}}{{{{{{{{{\boldsymbol{\nabla }}}}}}}}}}\cdot ({v}_{{{{{{{{{{\rm{ld}}}}}}}}}}}(\rho ){{{{{{{{{\boldsymbol{\nabla }}}}}}}}}}{v}_{{{{{{{{{{\rm{ld}}}}}}}}}}}(\rho )\rho ),$$

3 $$\dot{{{{{{{{{{\bf{v}}}}}}}}}}}+({{{{{{{{{\bf{v}}}}}}}}}}\cdot {{{{{{{{{\boldsymbol{\nabla }}}}}}}}}}){{{{{{{{{\bf{v}}}}}}}}}} {=} 	-\frac{1}{m}{{{{{{{{{\boldsymbol{\nabla }}}}}}}}}}({\;f}^{{\prime} }(\rho )-\kappa {{{{{{{{{{\boldsymbol{\nabla }}}}}}}}}}}^{2}\rho+\lambda {({{{{{{{{{\boldsymbol{\nabla }}}}}}}}}}\rho )}^{2}+{U}_{1}) \\ 	 -\gamma {{{{{{{{{\bf{v}}}}}}}}}}+\frac{{v}_{{{{{{{{{{\rm{ld}}}}}}}}}}}{(\rho )}^{2}}{\gamma }{{{{{{{{{{\boldsymbol{\nabla }}}}}}}}}}}^{2}{{{{{{{{{\rm{v}}}}}}}}}}+\frac{\xi }{m}({{{{{{{{{{\boldsymbol{\nabla }}}}}}}}}}}^{2}\rho ){{{{{{{{{\boldsymbol{\nabla }}}}}}}}}}\rho$$

with the velocity field **v**, the rotational diffusion coefficient *D*_R_, the free energy density *f*, its derivative ${f}^{{\prime} }={\partial }_{\rho }\,f$, the particle mass *m*, the friction coefficient *γ* = 1/(*mM*), and the local density-dependent swimming speed

4 $${v}_{{{{{{{{{{\rm{ld}}}}}}}}}}}(\rho )={v}_{0}-\frac{{A}_{1}}{\gamma m}\rho .$$

Here, *v*_0_ is the propulsion speed of a free particle and *A*_1_ is a constant (see Eq. (83)). We have also included an external potential *U*_1_ for generality. The form (4) agrees with the expression derived by Bickmann and Wittkowski^49^, who considered an overdamped system. In overdamped active matter, the existence of a density-dependent swimming speed—that can arise, e.g., from particle collisions in the case of active Brownian particles (ABPs) considered here or from quorum-sensing in the case of bacteria—is essential for the phenomenon of motility-induced phase separation (MIPS), where repulsively interacting particles phase-separate (which would not be possible in a passive system)^55^. From AMI+, we can see that *v*_ld_(*ρ*) plays two roles in the underdamped case: First, it leads to a second term in the continuity equation (2) in addition to the well-known passive term **∇**⋅(*ρ***v**). This second term is related to the self-propulsion term known from the active PFC model (see Methods). Second, it gives rise to an effective viscosity *v*_ld_(*ρ*)^2^/*γ*. This implies that a system of underdamped active particles should behave more like a viscous fluid for larger activity (larger *v*_ld_) and more like an ideal fluid for larger density (smaller *v*_ld_).

See Bär et al.^56^ for a discussion of other forms of effective viscosity in active matter.

AMI+ contains AMB+ as a limiting case. Showing this requires two approximations: First, we assume the system to be overdamped (large *γ*), i.e., we set the material derivative $\dot{{{{{{{{{{\bf{v}}}}}}}}}}}+({{{{{{{{{\bf{v}}}}}}}}}}\cdot {{{{{{{{{\boldsymbol{\nabla }}}}}}}}}}){{{{{{{{{\bf{v}}}}}}}}}}$ in Eq. (3) to zero, solve the resulting equation for **v** and insert the result into Eq. (2). (This is analogous to the procedure required for deriving overdamped from underdamped DDFT^41^). Second, using Eq. (4), we write in Eq. (2)

5 $$\frac{1}{2{D}_{{{{{{{{{{\rm{R}}}}}}}}}}}}{{{{{{{{{\boldsymbol{\nabla }}}}}}}}}}\cdot ({v}_{{{{{{{{{{\rm{ld}}}}}}}}}}}(\rho ){{{{{{{{{\boldsymbol{\nabla }}}}}}}}}}{v}_{{{{{{{{{{\rm{ld}}}}}}}}}}}(\rho )\rho )={{{{{{{{{\boldsymbol{\nabla }}}}}}}}}}\cdot (M\rho {{{{{{{{{\boldsymbol{\nabla }}}}}}}}}}{f}_{{{{{{{{{{\rm{e}}}}}}}}}}}^{{\prime} }(\rho ))+{{{{{{{{{\mathcal{O}}}}}}}}}}({\rho }^{3})$$

with the effective free energy density

6 $${f}_{{{{{{{{{{\rm{e}}}}}}}}}}}=\frac{1}{2M{D}_{{{{{{{{{{\rm{R}}}}}}}}}}}}\,\left({v}_{0}^{2}\rho \,\left(\ln ({\Lambda }^{2}\rho )-1\right)-\frac{3{v}_{0}{A}_{1}}{2\gamma m}{\rho }^{2}\right),$$

where *Λ* is the (irrelevant) thermal de Broglie wavelength, and then define *f*_o_ = *f* + *f*_e_ and ${f}_{{{{{{{{{{\rm{e}}}}}}}}}}}^{{\prime} }={\partial }_{\rho }\,{f}_{{{{{{{{{{\rm{e}}}}}}}}}}}$. Equation (6) shows that we can interpret ${v}_{0}^{2}/(2M{D}_{{{{{{{{{{\rm{R}}}}}}}}}}}{k}_{{{{{{{{{{\rm{B}}}}}}}}}}})$ with Boltzmann constant *k*_B_ as a shift of the temperature^57^, since the first term on the right-hand side has the form of an ideal gas free energy.

As shown in the Methods, the microscopic derivation reveals another form of effective temperature that is a feature of inertial active matter. The free energy density *f* appearing in Eq. (3) and microscopically determined by Eq. (111) in the Methods has, as a prefactor in the ideal gas term, a factor ${k}_{{{{{{{{{{\rm{B}}}}}}}}}}}T+m{v}_{0}^{2}/2$ with temperature *T* rather than *k*_B_*T* as in the passive case. This shows that the active kinetic energy $m{v}_{0}^{2}/2$ plays the role of a thermal energy in inertial active matter.

By taking the curl of Eq. (3) and defining the vorticity **ω** = **∇** × **v**, we can obtain the active vorticity equation

7 $$\dot{{{{{{{{{{\mathbf{\upomega }}}}}}}}}}}=-({{{{{{{{{\bf{v}}}}}}}}}}\cdot {{{{{{{{{\boldsymbol{\nabla }}}}}}}}}}){{{{{{{{{\mathbf{\upomega }}}}}}}}}}+({{{{{{{{{\mathbf{\upomega }}}}}}}}}}\cdot {{{{{{{{{\boldsymbol{\nabla }}}}}}}}}}){{{{{{{{{\bf{v}}}}}}}}}}-{{{{{{{{{\mathbf{\upomega }}}}}}}}}}({{{{{{{{{\boldsymbol{\nabla }}}}}}}}}}\cdot {{{{{{{{{\bf{v}}}}}}}}}})+\frac{{v}_{{{{{{{{{{\rm{ld}}}}}}}}}}}^{2}(\rho )}{\gamma }{{{{{{{{{{\boldsymbol{\nabla }}}}}}}}}}}^{2}{{{{{{{{{\mathbf{\upomega }}}}}}}}}}\\+\frac{1}{\gamma }\,\left({{{{{{{{{\boldsymbol{\nabla }}}}}}}}}}{v}_{{{{{{{{{{\rm{ld}}}}}}}}}}}^{2}(\rho )\right)\times {{{{{{{{{{\boldsymbol{\nabla }}}}}}}}}}}^{2}{{{{{{{{{\bf{v}}}}}}}}}}-\gamma {{{{{{{{{\mathbf{\upomega }}}}}}}}}}-\frac{\xi }{m}({{{{{{{{{\boldsymbol{\nabla }}}}}}}}}}\rho )\times ({{{{{{{{{\boldsymbol{\nabla }}}}}}}}}}{{{{{{{{{{\boldsymbol{\nabla }}}}}}}}}}}^{2}\rho ).$$

Starting from AMI+, we can again make two approximations: First, we assume that *v*_ld_(*ρ*) is small. From Eq. (4), we can see that this assumption is justified if *v*_0_ and *A*_1_ are both small (i.e., in the case of weak activity) or, for larger activities, if *v*_0_ ≈ *A*_1_*ρ*/(*γm*). Second, we drop the term proportional to *ξ*, such that the material derivative of **v** is given by the sum of the gradient of a generalized chemical potential and a damping term. Setting *ξ* = 0 is the usual approximation by which one gets from AMB+ to AMB.

We then obtain the simpler active model I (AMI), which is given by

8 $$\dot{\rho }=-{{{{{{{{{\boldsymbol{\nabla }}}}}}}}}}\cdot (\rho {{{{{{{{{\bf{v}}}}}}}}}}),$$

9 $$\dot{{{{{{{{{{\bf{v}}}}}}}}}}}+({{{{{{{{{\bf{v}}}}}}}}}}\cdot {{{{{{{{{\boldsymbol{\nabla }}}}}}}}}}){{{{{{{{{\bf{v}}}}}}}}}}=-\frac{1}{m}{{{{{{{{{\boldsymbol{\nabla }}}}}}}}}}({\;f}^{{\prime} }(\rho )-\kappa {{{{{{{{{{\boldsymbol{\nabla }}}}}}}}}}}^{2}\rho+\lambda {({{{{{{{{{\boldsymbol{\nabla }}}}}}}}}}\rho )}^{2}+{U}_{1})-\gamma {{{{{{{{{\bf{v}}}}}}}}}}.$$

Equation (9) can be written as

10 $$\dot{{{{{{{{{{\bf{v}}}}}}}}}}}+({{{{{{{{{\bf{v}}}}}}}}}}\cdot {{{{{{{{{\boldsymbol{\nabla }}}}}}}}}}){{{{{{{{{\bf{v}}}}}}}}}}=-\frac{1}{m}{{{{{{{{{\boldsymbol{\nabla }}}}}}}}}}\mu -\gamma {{{{{{{{{\bf{v}}}}}}}}}}$$

with a generalized chemical potential

11 $$\mu={f}^{{\prime} }(\rho )-\kappa {{{{{{{{{{\boldsymbol{\nabla }}}}}}}}}}}^{2}\rho+\lambda {({{{{{{{{{\boldsymbol{\nabla }}}}}}}}}}\rho )}^{2}+{U}_{1}.$$

It is straightforward to obtain AMB from AMI by taking the overdamped limit.

**Fig. 1:**
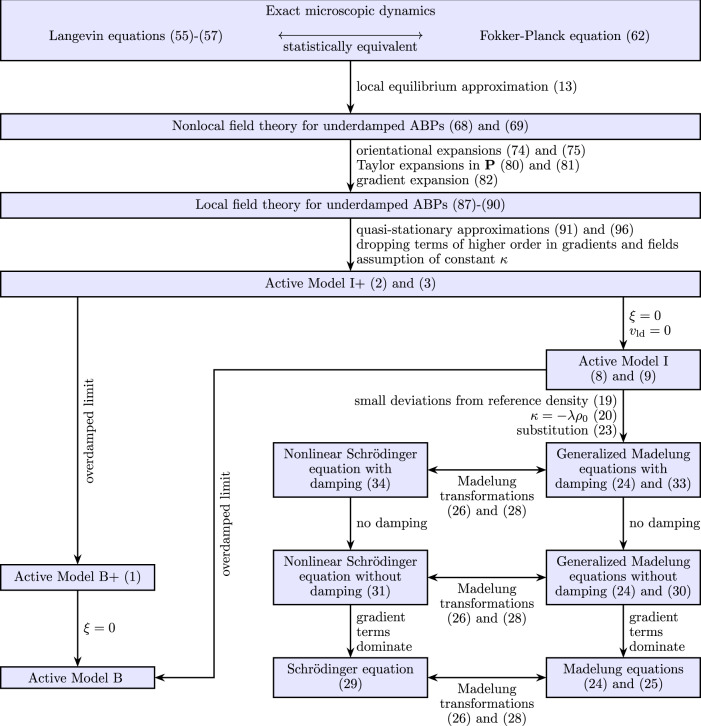
Illustration of the microscopic derivation and relations of the various models considered in this article. Starting from the microscopic dynamics of inertial active particles, a series of approximations leads to active models I and I+. More general models are obtained by omitting some approximations. Active models I and I+ contain an analog of the Schrödinger equation as well as the overdamped active models B and B+ as limiting cases.

AMI+ constitutes our first main result. The relations of the various models are illustrated in Fig. 1. This figure also shows the main steps of the microscopic derivation of AMI+, which is performed in the Methods.

### Mechanical vs thermodynamic velocity

Before we proceed with the main part of the discussion by deriving an analog of the Schrödinger equation from AMI, it is worth discussing two interesting features of the microscopic derivation of AMI+, namely its relation to the Mori-Zwanzig formalism and the fact that it implies a difference between two types of velocity. (The full microscopic derivation is shown in the Methods).

It is common in the theory of passive fluids, where the one-body distribution function *P*_1_ depends only on position **r** and momentum **p**, to apply the local equilibrium approximation^58,59^

12 $${P}_{1}({{{{{{{{{\bf{r}}}}}}}}}},{{{{{{{{{\bf{p}}}}}}}}}})=\frac{\rho ({{{{{{{{{\bf{r}}}}}}}}}})}{2\pi m{k}_{{{{{{{{{{\rm{B}}}}}}}}}}}T}\exp \left(-\frac{{({{{{{{{{{\bf{p}}}}}}}}}}-m{{{{{{{{{\bf{v}}}}}}}}}}({{{{{{{{{\bf{r}}}}}}}}}}))}^{2}}{2m{k}_{{{{{{{{{{\rm{B}}}}}}}}}}}T}\right).$$

The ansatz (12) (with *ρ*(**r**) replaced by an orientation-dependent density $\varrho ({{{{{{{{{\bf{r}}}}}}}}}},\hat{{{{{{{{{{\bf{u}}}}}}}}}}})$) has also been used in active matter physics^35^. Here, we apply the generalized local equilibrium approximation

13 $${P}_{1}({{{{{{{{{\bf{r}}}}}}}}}},{{{{{{{{{\bf{p}}}}}}}}}},\hat{{{{{{{{\bf {u}}}}}}}}})=\frac{\varrho ({{{{{{{{{\bf{r}}}}}}}}}},\hat{{{{{{{{{{\bf{u}}}}}}}}}}})}{2\pi m{k}_{{{{{{{{{{\rm{B}}}}}}}}}}}T}\exp \left(-\frac{{({{{{{{{{{\bf{p}}}}}}}}}}-m{\mathfrak{v}}({{{{{{{{{\bf{r}}}}}}}}}},\hat{{{{{{{{{{\bf{u}}}}}}}}}}}))}^{2}}{2m{k}_{{{{{{{{{{\rm{B}}}}}}}}}}}T}\right)$$

with a generalized velocity field ${\mathfrak{v}}({{{{{{{{{\bf{r}}}}}}}}}},\hat{{{{{{{{{{\bf{u}}}}}}}}}}})$ instead. (The motivation for this change is discussed below).

Using an ansatz of the form (13) (or (12) for a passive fluid) is required because the interaction-expansion method is (like many microscopic derivation methods) based on integrating an exact microscopic theory over the coordinates of all particles except for one, which leads to a dynamic equation for the order parameter fields that requires one or several closures. Equation (13) provides such a closure. We will now briefly discuss a different derivation method by which AMI+ can also be obtained, and explain how Eq. (13) can be understood in this formalism.

Among the most general methods for microscopic derivations is the Mori-Zwanzig formalism^60–64^, which allows one to obtain transport equations for an arbitrary set of slow variables by projecting the complete microscopic dynamics onto their subdynamics. This formalism can be applied also to active matter^5^ and thus represents an alternative route for a derivation of AMI+. In the Mori-Zwanzig formalism, one introduces a relevant distribution ${\bar{P}}_{N}$ that has the form^65,66^

14 $${\bar{P}}_{N}\propto \exp \left(-\frac{H-\mathop{\sum }\nolimits_{i=1}^{w}{a}_{i}^{\natural }{\widehat{A}}_{i}}{{k}_{{{{{{{{{{\rm{B}}}}}}}}}}}T}\right)$$

with the Hamiltonian *H*, the thermodynamic conjugates ${a}_{i}^{\natural }$ of the mean values *a*_*i*_ of the relevant variables ${\widehat{A}}_{i}$, and the number of relevant variables *w*. The Hamiltonian typically has the form $H=U(\{{{{{{{{{{{\bf{r}}}}}}}}}}}_{i}\})+\mathop{\sum }\nolimits_{i=1}^{N}{{{{{{{{{{\bf{p}}}}}}}}}}}_{i}^{2}/(2m)$ with the potential *U* and the position **r**_*i*_ and momentum **p**_*i*_ of the *i*-th particle. While Eq. (14) also has a local equilibrium form, it is not assumed that the actual *N*-particle distribution *P*_*N*_ actually looks like this^67^. In fact, calculating deviations of *P*_*N*_ from ${\bar{P}}_{N}$ is an essential part of the formalism^66^. The form (14) is chosen for information-theoretical reasons^64,68^, as it maximizes the informational entropy based on our macroscopically available knowledge^69^.

In fluid mechanics, one uses as a relevant variable the total momentum density operator $\widehat{{{{{{{{{{\bf{ g}}}}}}}}}}}({{{{{{{{{\bf{r}}}}}}}}}})=\mathop{\sum }\nolimits_{i=1}^{N}{{{{{{{{{{\bf{p}}}}}}}}}}}_{i}\delta ({{{{{{{{{\bf{r}}}}}}}}}}-{{{{{{{{{{\bf{r}}}}}}}}}}}_{i})$ with the Dirac delta distribution *δ* (**g** is the ensemble average of $\widehat{{{{{{{{{{\bf{g}}}}}}}}}}}$). Inserting ${\widehat {\bf A}}_{1}=\widehat{{{{{{{{{{\bf{g}}}}}}}}}}}$ into Eq. (14), writing **v** for **g**^*♮*^, and integrating over the phase-space coordinates of all except for one particle gives a distribution proportional to the distribution (12). Thus, the velocity field **v** is simply the thermodynamic conjugate for the momentum density^66^. In the active case, however, additional variables can become relevant. An essential parameter for active phase separation in overdamped^55^ and underdamped^27^ active fluids is the average of $\hat{{{{{{{{{{\bf{u}}}}}}}}}}}\cdot {{{{{{{{{\bf{p}}}}}}}}}}$ (which corresponds to the average of the projection of the particle momentum onto the direction of self-propulsion). Motivated by this observation, we use the momentum density polarization ${\underline{{{{\widehat{{{{{{{\boldsymbol{g}}}}}}}}}}}}}_{{{{{{{{{{\bf{P}}}}}}}}}}}({{{{{{{{{\bf{r}}}}}}}}}})=\mathop{\sum }\nolimits_{i=1}^{N}{\hat{{{{{{{{{{\bf{u}}}}}}}}}}}}_{i}\otimes {{{{{{{{{{\bf{p}}}}}}}}}}}_{i}\delta ({{{{{{{{{\bf{r}}}}}}}}}}-{{{{{{{{{{\bf{r}}}}}}}}}}}_{i})$ with the orientation vector of the *i*-th particle ${\hat{{{{{{{{{{\bf{u}}}}}}}}}}}}_{i}$ and the dyadic product ⊗ as a relevant variable in addition to $\widehat{{{{{{{{{{\bf{g}}}}}}}}}}}$. Using the same steps as before, Eq. (14) then gives the relevant one-body distribution function

15 $${\bar{P}}_{1}\propto \exp \left(-\frac{1}{{k}_{{{{{{{{{{\rm{B}}}}}}}}}}}T}\,\left(\frac{{{{{{{{{{{\bf{p}}}}}}}}}}}^{2}}{2m}-{{{{{{{{{\bf{v}}}}}}}}}}\cdot {{{{{{{{{\bf{p}}}}}}}}}}-{\underline{{{{{{{{{{\boldsymbol{v}}}}}}}}}}}}_{{{{{{{{{{\bf{P}}}}}}}}}}}:(\hat{{{{{{{{{{\bf{u}}}}}}}}}}}\otimes {{{{{{{{{\bf{p}}}}}}}}}})\right)\right),$$

where : denotes a double tensor contraction. Here, the local velocity polarization ${\underline{{{{{{{{{{\boldsymbol{v}}}}}}}}}}}}_{{{{{{{{{{\bf{P}}}}}}}}}}}$ is the thermodynamic conjugate for ${\underline{{{{\widehat{{{{{{{\boldsymbol{g}}}}}}}}}}}}}_{{{{{{{{{{\bf{P}}}}}}}}}}}$. The form (15) corresponds to our generalized local equilibrium form (13), as can be seen by inserting the orientational expansion (75) (see Methods) into Eq. (13). Note that Eq. (15) also explains why the density-dependent swimming speed (which comes from ${\underline{{{{{{{{{{\boldsymbol{v}}}}}}}}}}}}_{{{{{{{{{{\bf{P}}}}}}}}}}}$, see Methods) gives rise to an effective viscosity: Viscous terms arise from deviations of *P*_1_ from the form (12)^58^, and such a deviation arises here from activity in the form of ${\underline{{{{{{{{{{\boldsymbol{v}}}}}}}}}}}}_{{{{{{{{{{\bf{P}}}}}}}}}}}$. Moreover, since Eq. (15) reduces to the local equilibrium form (12) for ${\underline{{{{{{{{{{\boldsymbol{v}}}}}}}}}}}}_{{{{{{{{{{\bf{P}}}}}}}}}}}=\underline{{{{{{{{{{\boldsymbol{0}}}}}}}}}}}$, the term involving ${\underline{{{{{{{{{{\boldsymbol{v}}}}}}}}}}}}_{{{{{{{{{{\bf{P}}}}}}}}}}}$ accounts for the deviation of the active system from local equilibrium and detailed balance.

Note that differences between the relevant and the actual distribution (deviations from the generalized local equilibrium (13)) may lead to differences in the precise form of the transport equations by giving rise to additional viscous terms in the dynamic equation for ${\mathfrak{v}}$ (Eq. (69) in the Methods). In the interaction-expansion method, this could be incorporated by expanding a general distribution *P*_1_ around the distribution (13) (a procedure also employed in passive fluids^58^).

Why including an additional relevant variable related to activity is useful can be seen when analyzing what happens if it is not done, i.e., if we use the ansatz (12) instead of (13). In passive fluids, the local equilibrium approximation (12) can be rigorously justified because it arises as the zeroth-order expression in the Chapman-Enskog expansion^70^. The derivation for passive fluids uses the fact that the distribution (12) satisfies the requirement of detailed balance^70^. In active matter, however, detailed balance is violated^55^.

We can nevertheless try to use the ansatz (12) also in the active case, since it might still be a good approximation for small activities. Integrating the microscopic dynamics of *P*_1_ (Eq. (64) from the Methods) over **p** using Eq. (12) (with *ρ* replaced by *ϱ*) yields

16 $$\dot{\varrho }({{{{{{{{{\bf{r}}}}}}}}}},\hat{{{{{{{{{{\bf{u}}}}}}}}}}})=-{{{{{{{{{\boldsymbol{\nabla }}}}}}}}}}\cdot (\varrho ({{{{{{{{{\bf{r}}}}}}}}}},\hat{{{{{{{{{{\bf{u}}}}}}}}}}}){{{{{{{{{\bf{v}}}}}}}}}}({{{{{{{{{\bf{r}}}}}}}}}}))+{D}_{{{{{{{{{{\rm{R}}}}}}}}}}}{\partial }_{\varphi }^{2}\varrho ({{{{{{{{{\bf{r}}}}}}}}}},\hat{{{{{{{{{{\bf{u}}}}}}}}}}}).$$

A problem with the result (16) is that it is known from overdamped models^38,71^ that the governing equation for *ϱ* should contain a term $-{v}_{0}\hat{{{{{{{{{{\bf{u}}}}}}}}}}}\cdot {{{{{{{{{\boldsymbol{\nabla }}}}}}}}}}\varrho$ on the right-hand side which accounts for self-propulsion. Such a term cannot be obtained from Eq. (16) since **v** does not depend on $\hat{{{{{{{{{{\bf{u}}}}}}}}}}}$. To avoid this problem and to get an ansatz that has both the correct equilibrium and the correct overdamped limit, we use the generalized local equilibrium approximation (13) instead.

The seemingly minor difference between Eqs. (12) and (13) has profound implications for the definition of the velocity field **v**. In the theory of classical passive fluids, **v** can be defined in two ways:

First, there is the mechanical definition: The density *ρ* obeys a continuity equation ${\rm m}\dot{\rho }=-{{{{{{{{{\boldsymbol{\nabla }}}}}}}}}}\cdot {{{{{{{{{\bf{g}}}}}}}}}}$ with the momentum density **g**(**r**) = ∫ d^2^*p* **p***P*_1_(**r**,**p**). We can then define the velocity field as m**v** = **g**/*ρ*^69^, implying that

17 $$\dot{\rho }=-{{{{{{{{{\boldsymbol{\nabla }}}}}}}}}}\cdot (\rho {{{{{{{{{\bf{v}}}}}}}}}}).$$

Second, there is the thermodynamical definition: We use for the one-body distribution function *P*_1_ the local equilibrium form (12) and then define the velocity field to be the field **v** appearing in Eq. (12)^67^. Multiplying the dynamic equation for *P*_1_ by **p**, inserting Eq. (12), and integrating over **p** also leads to Eq. (17).

Since both definitions give the same result (17) in the passive case, their difference is usually not even mentioned. However, they are not equivalent for the active fluid considered here, which is assumed to be in a generalized local equilibrium of the form (13). While the mechanical route leads to Eq. (17) also in the active case, the thermodynamic route considered here gives the different result (2). Note that, since this difference arises both in the interaction-expansion method and in the Mori-Zwanzig formalism (where it is not assumed that the distribution actually has the form (13)), it is not an artifact of choosing the ansatz (13).

### Derivation of the Schrödinger equation

Now, we derive an equation that is formally equivalent to the Schrödinger equation from AMI given by Eqs. (8) and (9).

For this purpose, we assume ${f}^{{\prime} }(\rho )=0$ and *γ* = 0. (More precisely, since the microscopic model (Eqs. (55)–(61) from the Methods) becomes passive if *γ* is exactly zero, we assume a small but finite *γ* combined with a strong activity. A detailed discussion of this limit can be found in the Methods.) Then, Eq. (9) reads

18 $$\dot{{{{{{{{{{\bf{v}}}}}}}}}}}+({{{{{{{{{\bf{v}}}}}}}}}}\cdot {{{{{{{{{\boldsymbol{\nabla }}}}}}}}}}){{{{{{{{{\bf{v}}}}}}}}}}=-\frac{1}{m}{{{{{{{{{\boldsymbol{\nabla }}}}}}}}}}(-\kappa {{{{{{{{{{\boldsymbol{\nabla }}}}}}}}}}}^{2}\rho+\lambda {({{{{{{{{{\boldsymbol{\nabla }}}}}}}}}}\rho )}^{2}+{U}_{1}).$$

We define *ρ*_q_ = 2*ρ* (*ρ*_q_ will later be interpreted as the quantum-mechanical density) and assume that *ρ*_q_ has only small deviations from a spatially and temporally constant reference density *ρ*_0_. Noting that adding a constant to *ρ*_q_ has no influence on the dynamics of **v** once we have set ${f}^{{\prime} }=0$, we can then approximately write

19 $$\rho=\frac{1}{2}{\rho }_{{{{{{{{{{\rm{q}}}}}}}}}}}=\frac{{\rho }_{0}}{2}\frac{{\rho }_{{{{{{{{{{\rm{q}}}}}}}}}}}}{{\rho }_{0}}\; \,\approx\, \;\frac{{\rho }_{0}}{2}\ln \left(\frac{{\rho }_{{{{{{{{{{\rm{q}}}}}}}}}}}}{{\rho }_{0}}\right)+\,\,{{\mbox{irrelevant constant}}}\,.$$

As Eq. (8) is linear in *ρ*, it is left unchanged by the replacement *ρ* → *ρ*_q_/2, i.e., it holds for *ρ*_q_ in exactly the same way as for *ρ*. In Eq. (18), we replace *ρ* by ${\rho }_{0}\ln ({\rho }_{{{{{{{{{{\rm{q}}}}}}}}}}}/{\rho }_{0})/2$ (motivated by Eq. (19)) and assume

20 $$\kappa=-\lambda {\rho }_{0}.$$

This gives

21 $$\dot{{{{{{{{{{\bf{v}}}}}}}}}}}+({{{{{{{{{\bf{v}}}}}}}}}}\cdot {{{{{{{{{\boldsymbol{\nabla }}}}}}}}}}){{{{{{{{{\bf{v}}}}}}}}}}	=\frac{\kappa {\rho }_{0}}{2m}{{{{{{{{{\boldsymbol{\nabla }}}}}}}}}}\,\left({{{{{{{{{{\boldsymbol{\nabla }}}}}}}}}}}^{2}\ln \left(\frac{{\rho }_{{{{{{{{{{\rm{q}}}}}}}}}}}}{{\rho }_{0}}\right)+\frac{1}{2}{\,\,\left({{{{{{{{{\boldsymbol{\nabla }}}}}}}}}}\ln \left(\frac{{\rho }_{{{{{{{{{{\rm{q}}}}}}}}}}}}{{\rho }_{0}}\right)\right)}^{2}\right)-\frac{1}{m}{{{{{{{{{\boldsymbol{\nabla }}}}}}}}}}{U}_{1}\\ 	=\frac{1}{m}{{{{{{{{{\boldsymbol{\nabla }}}}}}}}}}\,\left(\kappa {\rho }_{0}\frac{{{{{{{{{{{\boldsymbol{\nabla }}}}}}}}}}}^{2}\sqrt{{\rho }_{{{{{{{{{{\rm{q}}}}}}}}}}}}}{\sqrt{{\rho }_{{{{{{{{{{\rm{q}}}}}}}}}}}}}-{U}_{1}\right).$$

The last step uses the identity^44^

22 $$\frac{{{{{{{{{{{\boldsymbol{\nabla }}}}}}}}}}}^{2}\vartheta }{\vartheta }={{{{{{{{{{\boldsymbol{\nabla }}}}}}}}}}}^{2}\ln (\vartheta )+{({{{{{{{{{\boldsymbol{\nabla }}}}}}}}}}\ln (\vartheta ))}^{2},$$

where *ϑ* is a function, and the fact that $\ln (\vartheta )=2\ln (\sqrt{\vartheta })$. Moreover, we set

23 $$\frac{{\hslash }^{2}}{2m}=\kappa {\rho }_{0}$$

with the reduced Planck constant *ℏ*. We then arrive at the Madelung equations^43^

24 $${\dot{\rho }}_{{{{{{{{{{\rm{q}}}}}}}}}}}=-{{{{{{{{{\boldsymbol{\nabla }}}}}}}}}}\cdot ({\rho }_{{{{{{{{{{\rm{q}}}}}}}}}}}{{{{{{{{{\bf{v}}}}}}}}}}),$$

25 $$\dot{{{{{{{{{{\bf{v}}}}}}}}}}}+({{{{{{{{{\bf{v}}}}}}}}}}\cdot {{{{{{{{{\boldsymbol{\nabla }}}}}}}}}}){{{{{{{{{\bf{v}}}}}}}}}}=\frac{1}{m}{{{{{{{{{\boldsymbol{\nabla }}}}}}}}}}\,\left(\frac{{\hslash }^{2}}{2m}\frac{{{{{{{{{{{\boldsymbol{\nabla }}}}}}}}}}}^{2}\sqrt{{\rho }_{{{{{{{{{{\rm{q}}}}}}}}}}}}}{\sqrt{{\rho }_{{{{{{{{{{\rm{q}}}}}}}}}}}}}-{U}_{1}\right).$$

Next, we assume that **v** is a potential flow such that we can write

26 $${{{{{{{{{\bf{v}}}}}}}}}}=\frac{1}{m}{{{{{{{{{\boldsymbol{\nabla }}}}}}}}}}S,$$

with a phase *S*, and that **v** satisfies the condition (see the article by Wallstrom^72^)

27 $$m{\oint }_{{{{{{{{{{\mathcal{L}}}}}}}}}}}{{{{{{{{{\rm{d}}}}}}}}}}{{{{{{{{{\bf{l}}}}}}}}}}\cdot {{{{{{{{{\bf{v}}}}}}}}}}=2\pi n\hslash$$

with a closed loop ${{{{{{{{{\mathcal{L}}}}}}}}}}$, a differential line element d**l**, and $n\in {\mathbb{Z}}$. We can then substitute

28 $$\psi=\sqrt{{\rho }_{{{{{{{{{{\rm{q}}}}}}}}}}}}{e}^{\frac{{{{{{{{{{\rm{i}}}}}}}}}}}{\hslash }S},$$

where *ψ* is (an analog of) the wave function and i is the imaginary unit. Combining Eqs. (24)–(26) and (28) then finally yields

29 $${{{{{{{{{\rm{i}}}}}}}}}}\hslash \dot{\psi }=-\frac{{\hslash }^{2}}{2m}{{{{{{{{{{\boldsymbol{\nabla }}}}}}}}}}}^{2}\psi+{U}_{1}\psi,$$

which is mathematically identical to the Schrödinger equation. The transformations required to obtain Eq. (29) from Eqs. (8) and (9) are summarized in Table 1. Equation (29) and its derivation from AMI+ constitute our second main result.

**Table 1:** Correspondences between variables and terms in AMI and in the Schrödinger equation

AMI (Eqs. (8) and (9))	Schrödinger equation (Eq. (29))	Relation
Particle density *ρ*	probability density *ρ*_q_ = ∣*ψ*∣^2^	*ρ*_q_ = 2*ρ*
Velocity **v**	phase of the wavefunction *S*	**v** = ${{{{{{{{{{\boldsymbol{\nabla }}}}}}}}}}}$*S*/*m*
Interaction/activity parameters *κ*, *λ*	reduced Planck constant *ℏ*	$\kappa {\rho }_{0}=-\lambda {\rho }_{0}^{2}={\hslash }^{2}/(2m)$
Generalized chemical potential *μ*	energy *E*	*μ* = *E*
Interaction/activity contributions − ${κ}{{{{{{{{{{\boldsymbol{\nabla }}}}}}}}}}}^{2}{\rho}+\lambda\,({{{{{{{{{{\boldsymbol{\nabla }}}}}}}}}}}{\rho})^{2}$	quantum potential $-({\hslash }^{2}/(2m))({{{{{{{{{{\boldsymbol{\nabla }}}}}}}}}}}^{2}\sqrt{{\rho }_{{{{{{{{{{\rm{q}}}}}}}}}}}})/\sqrt{{\rho }_{{{{{{{{{{\rm{q}}}}}}}}}}}}$	Derivable via $2\rho \approx {\rho }_{0}\ln ({\rho }_{{{{{{{{{{\rm{q}}}}}}}}}}}/{\rho }_{0})$

It is also instructive to see what happens if we do not set ${f}^{{\prime} }(\rho )=0$. In this case, Eq. (21) reads

30 $$\dot{{{{{{{{{{\bf{v}}}}}}}}}}}+({{{{{{{{{\bf{v}}}}}}}}}}\cdot {{{{{{{{{\boldsymbol{\nabla }}}}}}}}}}){{{{{{{{{\bf{v}}}}}}}}}}=\frac{1}{m}{{{{{{{{{\boldsymbol{\nabla }}}}}}}}}}\,\left(\kappa {\rho }_{0}\frac{{{{{{{{{{{\boldsymbol{\nabla }}}}}}}}}}}^{2}\sqrt{{\rho }_{{{{{{{{{{\rm{q}}}}}}}}}}}}}{\sqrt{{\rho }_{{{{{{{{{{\rm{q}}}}}}}}}}}}}-{f}^{{\prime} }\,\left(\frac{{\rho }_{{{{{{{{{{\rm{q}}}}}}}}}}}}{2}\right)-{U}_{1}\right).$$

Applying the substitutions (23), (26), and (28) then gives the nonlinear Schrödinger equation^73^

31 $${{{{{{{{{\rm{i}}}}}}}}}}\hslash \dot{\psi }=-\frac{{\hslash }^{2}}{2m}{{{{{{{{{{\boldsymbol{\nabla }}}}}}}}}}}^{2}\psi+{U}_{1}\psi+{f}^{{\prime} }\,\left(\frac{|\psi {|}^{2}}{2}\right)\psi .$$

For example, if we set ${f}^{{\prime} }=a{\rho }_{{{{{{{{{{\rm{q}}}}}}}}}}}$ with a constant *a*, we find^74^

32 $${{{{{{{{{\rm{i}}}}}}}}}}\hslash \dot{\psi }=-\frac{{\hslash }^{2}}{2m}{{{{{{{{{{\boldsymbol{\nabla }}}}}}}}}}}^{2}\psi+{U}_{1}\psi+a|\psi {|}^{2}\psi,$$

which is the Gross-Pitaevskii equation. This equation has a wide range of applications, such as modeling Bose-Einstein condensates (see the article by Mocz and Succi^74^ and references therein).

In the considered active matter system, an even more realistic case would be to also have *γ* ≠ 0. In this case, Eq. (21) reads

33 $$\dot{{{{{{{{{{\bf{v}}}}}}}}}}}+({{{{{{{{{\bf{v}}}}}}}}}}\cdot {{{{{{{{{\boldsymbol{\nabla }}}}}}}}}}){{{{{{{{{\bf{v}}}}}}}}}}=\frac{1}{m}{{{{{{{{{\boldsymbol{\nabla }}}}}}}}}}\,\left(\kappa {\rho }_{0}\frac{{{{{{{{{{{\boldsymbol{\nabla }}}}}}}}}}}^{2}\sqrt{{\rho }_{{{{{{{{{{\rm{q}}}}}}}}}}}}}{\sqrt{{\rho }_{{{{{{{{{{\rm{q}}}}}}}}}}}}}-{f}^{{\prime} }\,\left(\frac{{\rho }_{{{{{{{{{{\rm{q}}}}}}}}}}}}{2}\right)-{U}_{1}\right)-\gamma {{{{{{{{{\bf{v}}}}}}}}}}.$$

The Madelung transformations then lead to^75^

34 $${{{{{{{{{\rm{i}}}}}}}}}}\hslash \dot{\psi }=-\frac{{\hslash }^{2}}{2m}{{{{{{{{{{\boldsymbol{\nabla }}}}}}}}}}}^{2}\psi+{f}^{{\prime} }\,\left(\frac{|\psi {|}^{2}}{2}\right)\psi+{U}_{1}\psi -\frac{{{{{{{{{{\rm{i}}}}}}}}}}\hslash \gamma }{2}\ln \left(\frac{\psi }{{\psi }^{\star }}\right)\psi,$$

which is the Schrödinger-Langevin equation^76^ (transformed into local form, without the noise term, and with an additional nonlinear term ${f}^{{\prime} }\psi$).

The derivation of the (analog of the) Schrödinger equation (29) and its generalizations (31) and (34) from AMI is visualized in Fig. 1.

### Physical significance of the active-quantum mapping

Having established the mathematical relation between AMI and the Schrödinger equation, we now discuss the physical significance of the active-quantum mapping.

Mathematically, the fact that AMI allows one to derive the Madelung equations is essentially a consequence of the fact that AMI is a compressible Euler equation with the pressure being given by the most general expression of a certain order in gradients and densities. Since the Madelung equations are of this order in gradients and densities (if we can approximate densities by their logarithm), they must be contained in AMI. Moreover, we cannot use AMI+ since it leads to velocity fields with non-vanishing rotation, which does not make sense if we want to interpret the velocity as the gradient of a phase. In the overdamped case, it has been shown that the rotational terms constituting the difference between AMB and AMB+ are not relevant for quorum-sensing bacteria^20^, which therefore constitute a promising model system for our purposes. To obtain a more general model, we could also have included the terms $-{{{{{{{{{\boldsymbol{\nabla }}}}}}}}}}{f}^{{\prime} }(\rho )/m$ (with ${f}^{{\prime} }(\rho )={k}_{{{{{{{{{{\rm{B}}}}}}}}}}}T\ln ({\Lambda }^{2}\rho )$), −*γ***v**, and ${v}_{{{{{{{{{{\rm{ld}}}}}}}}}}}^{2}(\rho ){{{{{{{{{{\boldsymbol{\nabla }}}}}}}}}}}^{2}{{{{{{{{{\bf{v}}}}}}}}}}/\gamma$ from AMI+. In this case, we would have obtained a model similiar to the isothermal quantum Navier-Stokes equation, where the viscosity also depends on the density (although in a different way)^77^. Of course, this viscosity has a different physical interpretation since it does not arise from a density-dependent swimming speed. Instead, the viscous terms are obtained in the standard way from a Chapman-Enskog expansion^77^.

It should be noted that, strictly speaking, we have not derived the Schrödinger equation (since quantum physics is not a description of the dynamics of active classical particles), but an active field theory that has the same form as the Schrödinger equation. This is important for the physical interpretation. If we want to think of Eq. (29) as a limiting case of AMI, then ∣*ψ*∣^2^ is proportional to the particle density of a classical many-body system. In contrast, if we think of Eq. (29) as the quantum-mechanical Schrödinger equation, then ∣*ψ*∣^2^ is the probability density of a single quantum-mechanical particle.

Equation (25) contains a term $({\hslash }^{2}/(2{m}^{2})){{{{{{{{{\boldsymbol{\nabla }}}}}}}}}}(({{{{{{{{{{\boldsymbol{\nabla }}}}}}}}}}}^{2}\sqrt{{\rho }_{{{{{{{{{{\rm{q}}}}}}}}}}}})/\sqrt{{\rho }_{{{{{{{{{{\rm{q}}}}}}}}}}}})$—proportional to the gradient of the quantum potential $-({\hslash }^{2}/(2m))({{{{{{{{{{\boldsymbol{\nabla }}}}}}}}}}}^{2}\sqrt{{\rho }_{{{{{{{{{{\rm{q}}}}}}}}}}}})/\sqrt{{\rho }_{{{{{{{{{{\rm{q}}}}}}}}}}}}$, which gives rise to a quantum pressure^78^—on the right-hand side. This term, which disappears in the classical limit *ℏ* → 0, translates into the term − (*ℏ*^2^/(2*m*))**∇**^2^*ψ* in Eq. (29) after the transformations (26) and (28). From a quantum-mechanical point of view, this term arises from the momentum operator appearing in the kinetic part of the quantum Hamiltonian. From a classical point of view, however, this term arises from the terms − *κ***∇**^2^*ρ* + *λ*(**∇***ρ*)^2^ in Eq. (9) (which come from activity and interactions), combined with the assumption (20). We will later demonstrate numerically that this assumption can also be relaxed. Consequently, the classical limit in quantum mechanics corresponds to the passive noninteracting limit in active matter.

The usefulness of this mapping lies in two aspects. First, the analogy to soft matter can be used to better understand effects that are associated with the Schrödinger equation. This will be illustrated below using the example of dark matter. Second, we can reproduce effects known from quantum mechanics, which arise as a consequence of the quantum potential, in a classical soft matter system, where they arise from activity and/or interactions. In particular, our knowledge about the numerous quantum-mechanical phenomena that arise from the interplay with external fields can be used to better understand the behavior of active matter in external fields, a topic currently of high interest^50^. As an example, we will later use the mapping to find an active analog of the tunnel effect.

### Analogy to dark matter

In this section, we use the active-quantum mapping derived above to establish a relation between inertial active matter and dark matter. This illustrates the usefulness of this mapping, as it shows that active matter can be used as a model for dark matter (for example in analog experiments) and that our understanding of pattern formation can become useful for astrophysics.

An important field of application for the Madelung equations is the study of dark matter^78^. Recently, there has been an increase of interest in so-called fuzzy dark matter (FDM), which consists of ultralight scalar particles. It was introduced to avoid the problem that the standard cold dark matter models predicted cuspy halos and excessive small-scale structures, in conflict with observations^79,80^. Further motivations for research on FDM are the lack of evidence for other dark matter candidates and the fact that such ultralight particles are predicted by various models from particle physics (such as string theory)^81^.

On galactic scales, one can neglect self-interactions of the real scalar field representing dark matter and use a simple quadratic action functional^82^. In the nonrelativistic limit, the real scalar field can be re-written using a complex field *ψ* that obeys a Schrödinger equation with modifications accounting for cosmic expansion^81^. These modifications can be neglected on galactic scales^82^ and are also neglected here. The FDM particles are mostly in the ground state and can thus be described by a single macroscopic wavefunction as in a Bose-Einstein condensate^81^. It is very common in dark matter physics to transform from the Schrödinger equation to the Madelung equations since this allows to use hydrodynamic codes^82^. FDM can then be described by the Madelung equations (24) and (25) coupled to the Poisson equation^83^

35 $${{{{{{{{{{\boldsymbol{\nabla }}}}}}}}}}}^{2}{U}_{1}=4\pi G{m}^{2}{\rho }_{{{{{{{{{{\rm{q}}}}}}}}}}}$$

with the gravitational constant *G*. Equation (35) determines *U*_1_, which is here the gravitational potential, via the density *ρ*_q_.

Let us now consider the dynamics of a system of ABPs with an electric charge *q*. Its dynamics would be given by AMI in the limit where *v*_ld_ and *ξ* are small. The (electrostatic) potential *U*_1_ could be calculated from the charge distribution *ρ* via the Poisson equation

36 $${{{{{{{{{{\boldsymbol{\nabla }}}}}}}}}}}^{2}{U}_{1}=-\frac{{q}^{2}}{\epsilon }\rho$$

with the permittivity *ϵ*.

As shown above, AMI contains the Madelung equations as a limiting case. Therefore, in the quantum limit, an underdamped charged active matter system would be described by equations of the same form as a fuzzy dark matter system, suggesting an interesting parallel between active and astrophysical systems.

The analogy between dark and active matter is further supported by the fact that (as mentioned above) fuzzy dark matter models are based on Bose-Einstein condensates, which have been found also in active matter^1–3^. Also, the full governing equations for dark matter contain an additional nonlinearity as in Eq. (32)^78^, just as the full governing equations for active matter contain an additional nonlinearity due to the nonzero function ${f}^{{\prime} }$. These nonlinearities have a different physical interpretation in the two contexts. If Eq. (31) is applied to active matter, the function ${f}^{{\prime} }$ primarily arises from the temperature (see Eq. (111)). In FDM, however, it would describe self-interactions^78^.

Note, however, that there is also an important difference, namely the fact that the density appears with a different sign in the gravitational Poisson equation (35) and the electrostatic Poisson equation (36). This is a consequence of the fact that gravity is a purely attractive force, whereas electrostatic forces are repulsive for particles of the same charge. Therefore, the patterns observed in fuzzy dark matter and in charged active systems might be quite different. Schrödinger equations coupled to electrostatic Poisson equations of the form (36) are used in the theory of quantum plasmas^84^. Note that both dark matter^85^ and quantum plasmas^86^ can be found to exhibit solitonic solutions in a Schrödinger-Poisson model, such that solitons are likely to be observed also in charged inertial active matter.

When comparing the use of Eq. (29) in standard quantum mechanics and in FDM, two important differences should be noted. First, in the context of FDM, *ψ* describes the density of a many-body system, not of a single particle. Second, Eq. (29) is only approximately valid for dark matter, both due to cosmic expansion^81^ and due to the presence of nonlinear terms as in Eq. (32). Notably, FDM has in common with active matter both the many-body interpretation and the larger complexity of the actual governing equations.

FDM constitutes an important example for a system where the mapping to a soft matter system can contribute to a better physical understanding of the Schrödinger equation. Recall that FDM was introduced because existing dark matter models predict excessive small-scale structures^80^. The suppression of small-scale structure in FDM is a consequence of quantum pressure^87^. In our mapping, the quantum pressure corresponds to the gradient terms in the chemical potential (11). This result gives a good physical intuition for why FDM does better than older dark matter models regarding the small-scale problem: It is well known that gradient terms in the chemical potential suppress the formation of small-scale structure, since they (if they have the right sign) lead to an energetic penalty for interfaces. Using the active-quantum mapping developed here, such standard insights from pattern formation theory in soft matter physics can become fruitful also for astrophysics. In particular, knowledge of pattern formation effects from soft matter can be used to develop more sophisticated models for astrophysical pattern formation.

### Tunnel effect

After having introduced the general theories AMI and AMI+ and establishing a mapping between AMI and the Schrödinger equation, we now turn to an application of this mapping by deriving and investigating an active analogon of the tunnel effect. In this section, we restrict ourselves to one-dimensional systems.

Time-independent problems in quantum mechanics can be described by the stationary Schrödinger equation

37 $$E\psi=-\frac{{\hslash }^{2}}{2m}{\partial }_{x}^{2}\psi+{U}_{1}\psi$$

with the energy *E*. A central phenomenon of quantum mechanics is the tunnel effect, where a particle has non-zero probability of traveling through a potential barrier that it could not pass through classically. It can be described theoretically by solving Eq. (37) for the potential

38 $${U}_{1}(x)=\,\left\{\begin{array}{ll}0\quad &\,{{\mbox{for}}}\,\,x \, < -L,\hfill\\ {V}_{0}\quad &\,{{\mbox{for}}}\,\,-L\,\le\, x \,\le\, L,\\ 0\quad &\,{{\mbox{for}}}\,\,x \, > \, L,\hfill\end{array}\right.$$

where *V*_0_ is the height and 2*L* the width of the potential barrier. As is well known, the solution of Eq. (37) with the potential (38) is given by

39 $$\psi (x)=\,\left\{\begin{array}{ll}{e}^{{{{{{{{{{\rm{i}}}}}}}}}}kx}+{R}_{1}{e}^{-{{{{{{{{{\rm{i}}}}}}}}}}kx}\quad &\,{{\mbox{for}}}\,\,x \, < -L,\hfill\\ {T}_{2}{e}^{-\varkappa x}+{R}_{2}{e}^{\varkappa x}\quad &\,{{\mbox{for}}}\,\,-L\,\le\, x\,\le\, L,\\ {T}_{3}{e}^{{{{{{{{{{\rm{i}}}}}}}}}}kx}\hfill\quad &\,{{\mbox{for}}}\,\,x \, > \, L \hfill\end{array}\right.$$

with the wavenumbers

40 $$k=\sqrt{\frac{2mE}{{\hslash }^{2}}},$$

41 $$\varkappa=\sqrt{\frac{2m({V}_{0}-E)}{{\hslash }^{2}}},$$

the transmission coefficients *T*_2_ and *T*_3_, and the reflection coefficients *R*_1_ and *R*_2_. (Explicit expressions for these coefficients are given in the article by Heifetz and Plochotnikov^88^). The physical interpretation of the solution (39) is that it describes the wavefunction of a particle with energy *E* that approaches a rectangular potential barrier of height *V*_0_ > *E* from the left, decays within the barrier, and continues to travel as a wave on the right of the barrier. The density *ρ*_q_(*x*) = ∣*ψ*(*x*)∣^2^ gives the probability that the particle is found at a certain position *x* in a position measurement. Since this probability is non-zero for *x* > *L*, there is a non-zero probability that the particle passes through a barrier that it could not have passed through classically. This phenomenon is known as the tunnel effect.

Due to the linearity of Eq. (37), another solution is given by

42 $$\psi (x)=\,\left\{\begin{array}{ll}\frac{1}{\sqrt{2}}({e}^{{{{{{{{{{\rm{i}}}}}}}}}}kx}+({R}_{1}+{T}_{3}){e}^{-{{{{{{{{{\rm{i}}}}}}}}}}kx})\quad &\,{{\mbox{for}}}\,\,x \, < -L,\hfill\\ \frac{1}{\sqrt{2}}({T}_{2}+{R}_{2})({e}^{-\varkappa x}+{e}^{\varkappa x})\quad &\,{{\mbox{for}}}\,\,-L\,\le\, x\,\le\, L,\\ \frac{1}{\sqrt{2}}({e}^{-{{{{{{{{{\rm{i}}}}}}}}}}kx}+({R}_{1}+{T}_{3}){e}^{{{{{{{{{{\rm{i}}}}}}}}}}kx})\quad &\,{{\mbox{for}}}\,\,x \, > \, L,\hfill\end{array}\right.$$

which is simply the superposition of the solution given by Eq. (39) and the same solution mirror reflected at *x* = 0 (corresponding to a particle coming from the right). Such a symmetric tunneling solution has advantages in a numerical treatment (as it allows to use periodic boundary conditions) and captures the same physics. The quantum-mechanical density *ρ*_q_ = ∣*ψ*∣^2^ for the solution (42) is given by

43 $${\rho }_{{{{{{{{{{\rm{q}}}}}}}}}}}(x)=\,\left\{\begin{array}{ll}\frac{1}{2}(1+{R}^{2}+2R\cos (2kx-\alpha ))\quad &\,{{\mbox{for}}}\,\,x \, < -L,\hfill\\|{T}_{2}+{R}_{2}{|}^{2}(\cosh (2\varkappa x)+1)\quad \hfill&\,{{\mbox{for}}}\,\,-L\,\le\, x\,\le\, L,\\ \frac{1}{2}(1+{R}^{2}+2R\cos (2kx+\alpha ))\quad &\,{{\mbox{for}}}\,\,x \, > \, L,\hfill\end{array}\right.$$

where we have written *R*_1_ + *T*_3_ = *Re*^i*α*^ with the modulus *R* and the phase *α* of the complex number *R*_1_ + *T*_3_.

Using the Madelung transform, the nondimensionalized form of Eq. (37) for *v* = 0 reads (see Methods)

44 $$E=-\frac{{\hslash }^{2}}{2m}\,\left(\frac{1}{2}{\partial }_{x}^{2}\ln ({\rho }_{{{{{{{{{{\rm{q}}}}}}}}}}})+\frac{1}{4}{({\partial }_{x}\ln ({\rho }_{{{{{{{{{{\rm{q}}}}}}}}}}}))}^{2}\right)+{U}_{1}.$$

We now show how an analogon of the tunnel effect can be found in active matter. For simplicity, we set *v* = 0, such that AMI reduces to *μ* = const. with *μ* given by Eq. (11). Solutions of Eq. (11) with *μ* = const. are also stationary solutions of AMB, such that all of the following considerations apply to both AMI and AMB.

A nondimensionalization (see Methods) gives

45 $$\mu={f}^{{\prime} }(\rho )-\kappa {\partial }_{x}^{2}\rho+\lambda {({\partial }_{x}\rho )}^{2}+{U}_{1},$$

which is simply Eq. (11) in dimensionless form.

We now consider the special case with *κ* = − *λ* and ${f}^{{\prime} }=0$, in which Eq. (45) reduces to

46 $$\mu=-\kappa ({\partial }_{x}^{2}\rho+{({\partial }_{x}\rho )}^{2})+{U}_{1},$$

A solution of Eq. (46) for the potential (38) is given by

47 $$\rho (x)=\,\left\{\begin{array}{ll}\ln (\cos (k(x+L)+\alpha ))+A\quad \hfill&\,{{\mbox{for}}}\,\,x \, < -L,\hfill\\ \ln (\cosh (\varkappa x))+B\quad \hfill&\,{{\mbox{for}}}\,\,-L\,\le\, x\,\le\, L,\\ \ln (\cos (k(x-L)-\alpha ))+A\quad &\,{{\mbox{for}}}\,\,x \, > \, L\hfill\end{array}\right.$$

with the wavenumbers

48 $$k=\sqrt{\frac{\mu }{\kappa }},$$

49 $$\varkappa=\sqrt{\frac{{V}_{0}-\mu }{\kappa }},$$

the phase shift

50 $$\alpha=\arctan \left(\frac{\varkappa }{k}\tanh (\varkappa L)\right),$$

and two constants *A* and *B* that satisfy

51 $$A-B=\ln (\cosh (\varkappa L))-\ln (\cos (\alpha )).$$

Equations (50) and (51) ensure that *ρ* and ∂_*x*_*ρ* are continuous at the boundaries of the potential barrier. We have thus found an analytical solution of Eq. (46) for an active system at a potential barrier, namely Eq. (47). At the boundary of the potential barrier, a discontinuity in $\kappa {\partial }_{x}^{2}\rho$ balances the discontinuity in *U*_1_ and thereby ensures that *μ* is constant (as required for a stationary solution). Following the analysis by Heifetz and Plochotnikov^88^, we can define $I=\lambda {({\partial }_{x}\rho )}^{2}$ (which is here the active term) and a pressure $\Pi=-\rho \kappa {\partial }_{x}^{2}\rho$. Π is the pressure one would get from the thermodynamic expression *μρ* − *f* ^14^ for *λ* = 0 and *f* = 0. (Here, we have *λ* ≠ 0, so Π is in general not equal to the thermodynamic or mechanical pressure in the active system.) With these definitions, Eq. (45) gives for ${f}^{{\prime} }=0$

52 $$\mu=\frac{\Pi }{\rho }+I+{U}_{1}.$$

At the boundaries of the potential barrier, *ρ* and *I* are continuous. The tunneling is thus a consequence of a pressure jump ΔΠ = *ρV*_0_ at the boundaries.

The stationary Madelung equation (44) coincides with the stationary form of AMI or AMB given by Eq. (46) if we identify $\rho=\ln ({\rho }_{{{{{{{{{{\rm{q}}}}}}}}}}})/2$ (cf. Eq. (19)), *κ* = *ℏ*^2^/(2*m*) (cf. Eq. (23)), and *μ* = *E*. Therefore, we do not even need to employ the approximation (19) from the dynamical case, we can just straightforwardly map the quantum onto the classical problem. Taking the logarithm of the quantum solution (43) does indeed give us something that (apart from phases and prefactors) looks like Eq. (47), indicating that similar physical mechanisms act here. In particular, the change in the potential leads to a shift in the wavenumber from *k* to ϰ that gives a density decay within the barrier for *μ* < *V*_0_ (or *E* < *V*_0_) both in the quantum and in the active case (compare Eqs. (40) and (41) to Eqs. (48) and (49)).

### Numerical continuation

The strong mathematical analogy between AMI and the Madelung equations (or between AMB and the stationary Schrödinger equation) holds only for the rather special case ${f}^{{\prime} }(\rho )=0$ and *κ* = −*λ*. In a real experiment, these equalities will, of course, be realized at most approximately. Therefore, it is investigated in this section how robust the analogy between active matter and quantum mechanics is if these equalities are modified.

For this purpose, we consider the more general model

53 $$\mu=a\rho -\kappa {\partial }_{x}^{2}\rho+\lambda {({\partial }_{x}\rho )}^{2}+{U}_{1},$$

where *U*_1_ is still given by Eq. (38). For *a* = 0 and *κ* = −*λ*, the analytical solution (47) is known. Starting from these parameter values and this solution, we can find solutions for Eq. (53) for other parameter values via numerical continuation (see Methods).

We wish to ensure that the density *ρ* is always positive and that *ρ* and ∂_*x*_*ρ* take identical values on both boundaries of the domain, allowing us to use periodic boundary conditions. This determines the one-dimensional domain Ω = [ − *α*/*k* − *L*, *L* + *α*/*k*]. Furthermore, we set *κ* = 1, *V*_0_ = 5, *μ* = 1, and *α* = *π*/4 and use the analytical solution (47) as starting solution for the continuation. Using Eqs. (48)–(50) we obtain $L={{{{{{{{{\rm{arctanh}}}}}}}}}}(1/2)/2$. Moreover, we set *B* = 0.5 (an arbitrary positive constant can be chosen here); *A* is then determined by Eq. (51). Note that with this also the averaged rescaled particle density is determined as $\bar{\rho }={\int}_{\Omega }{{{{{{{{{\rm{d}}}}}}}}}}x\rho (x)/|{{\Omega}}|\,\approx\, 0.7945$ (where ∣Ω∣ is the domain length). It can be chosen arbitrarily using different values of *B*. Hence, the following result does not depend on the particle number. The starting state is now continued, changing various parameters while keeping $\bar{\rho }$ fixed. This in turn determines *μ* as corresponding Lagrange multiplier. Alternatively, one could keep *μ* fix, in which case $\bar{\rho }$ would change during the continuation. However, this is not pursued here.

**Fig. 2:**
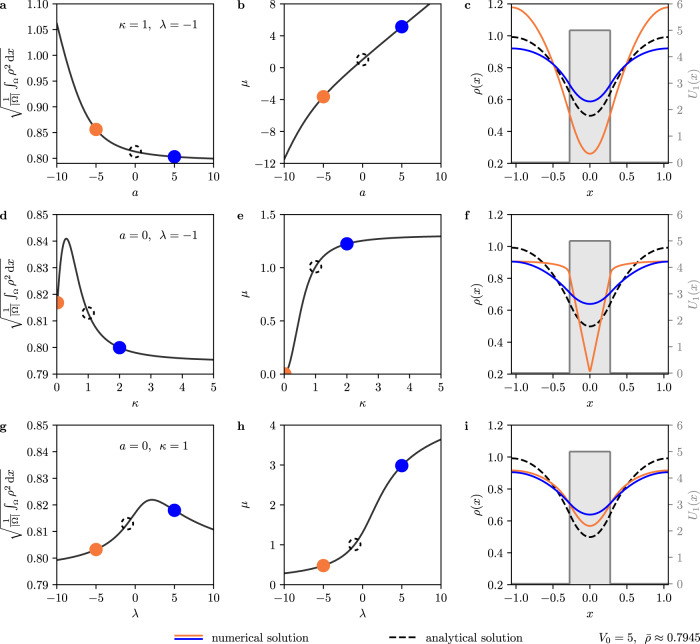
Results of the numerical continuation of Eq. (53). **a–c** Numerical continuation with control parameter *a* with fixed parameter values *κ* = − *λ* = 1. The plots show **a** the L_2_-norm of *ρ* as a function of *a*, **b** the chemical potential *μ* as a function of *a*, and **c** density profiles for selected parameter values as indicated by circles of corresponding colors in **a** and **b**, compared to the analytical solution (47) (dashed curve), which is used as the starting point for the continuation. **d–f** Like **a**–**c**, but with varying *κ* and fixed *a* = 0 and *λ* = − 1. **g**–**i** Like **a–c**, but with varying *λ* and fixed *a* = 0 and *κ* = 1. Note that the general form of the analytical solution persists also for other parameter values, indicating that the tunnel effect in model (53) is a robust phenomenon.

Figure 2 shows bifurcation diagrams and solution profiles that illustrate the tunnel effect that can be observed in model (53). In Fig. 2a, d, g and Fig. 2b, e, h we see how the L_2_-norm $\sqrt{{\int}_{\Omega }{{{{{{{{{\rm{d}}}}}}}}}}x{\rho }^{2}(x)/|\Omega|}$ and the generalized chemical potential *μ*, respectively, depend on the parameters (panels a, b) *a*, (panels d, e) *κ*, and (panels g, h) *λ*. Finally, Fig. 2c, f, i present solution profiles for the states indicated by orange and blue circles in the corresponding bifurcation diagrams to their left. The dashed black curve in each solution plot indicates the analytical solution given by Eq. (47) for comparison.

The solution profiles show that the general form does not change significantly if the parameter values are not exactly those used for the analytical mapping. This indicates that the tunnel effect, and the general active-quantum analogy presented here, are not an artifact of picking the parameter values in such a way that it works, but rather a robust phenomenon that can be investigated also in microscopic simulations and experiments. Furthermore, according to a linear stability analysis that is performed during the continuation (see Methods), the solution is linearly stable with respect to perturbations compatible with mass conservation for all considered parameter values. Despite this limitation and the fact that we consider a small domain, the stability of all solutions emphasizes the relevance for experiments.

We can also get a more detailed idea of the effect that changing the various parameters has on the solution (47). In general, a steep decrease of *ρ* towards *x* = 0 indicates that the field cannot penetrate far into the potential barrier, whereas a more flat curve is a sign of a strong tunnel effect. Changing *λ* has no strong effect on the form of the solution (Fig. 2i). The tunnel effect becomes more pronounced for positive values of *a*, whereas it is suppressed by negative ones (Fig. 2c). Since positive values of *a* are more plausible on physical grounds (one would typically expand *f* around a local minimum rather than around a local maximum), we can expect this tunneling to be even more significant in real systems. Note that for sufficiently large values of *a*, we get *μ* > *V*_0_, such that strictly speaking we do not have tunneling anymore (since tunneling requires *E* < *V*_0_ and *μ* corresponds to *E*). For *μ* > *V*_0_, ϰ becomes imaginary (see Eq. (49)) such that *ρ* has the form $\ln (\cos (x))$ also within the barrier. The strongest effect can be found by varying *κ* (Fig. 2f). If it is small (close to zero), we observe a sharp decrease and thus very weak tunneling. For larger *κ*, on the other hand, the field can pass through the barrier much more easily. This result is plausible since, as indicated above, it is the discontinuity in the *κ* term that balances the discontinuity of the potential. Also, larger values of *κ* imply that gradients, which are smaller if the fluid passes through the barrier (i.e., if the tunnel effect is present), are associated with an energetic cost, implying that tunneling is more likely to occur for larger *κ*.

### Physical interpretation of the active tunnel effect

Finally, we turn to a physical discussion of the active tunnel effect.

**Fig. 3:**
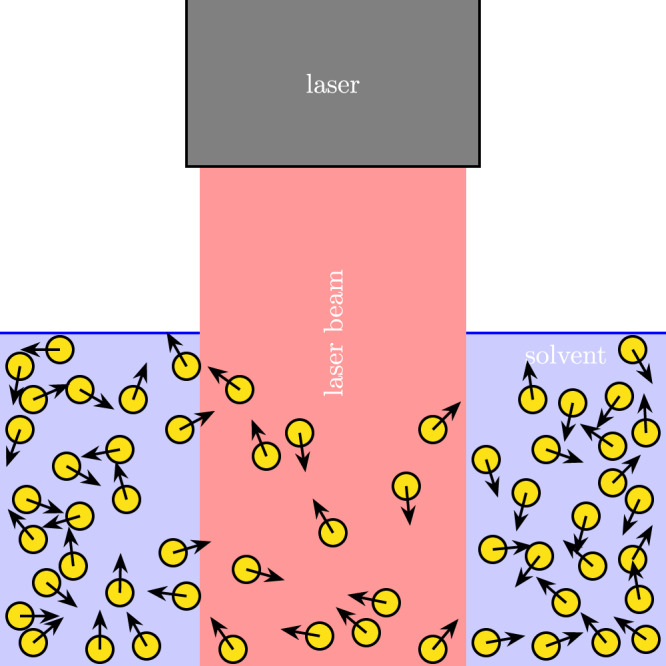
Possible experimental realization of the active tunnel effect. Active dielectric spheres (with orientations indicated by arrows) are immersed in a solvent and illuminated by a laser beam with rectangular intensity profile. The intensity gradient at the boundaries of the beam gives rise to a force pushing the particles outwards. Due to activity and interactions, the density decays smoothly at the beam boundaries, i.e., it is low also in the vicinity of the beam and not only in the illuminated area.

A first question that is relevant here is what one has to look at if one wants to see this effect, i.e., what a possible experimental realization could be. While many realizations are conceivable, we turn for concreteness to a system of dielectric spherical (active) particles with effective polarizability *p*_0_ illuminated by a laser beam with intensity *I*_laser_(**r**), giving rise to a gradient force ${{{{{{{{{{\bf{F}}}}}}}}}}}_{{{{{{{{{{\rm{grad}}}}}}}}}}}={p}_{0}{{{{{{{{{\boldsymbol{\nabla }}}}}}}}}}{I}_{{{{{{{{{{\rm{laser}}}}}}}}}}}/4$^89^ and thereby to a potential *U*_1_ = − *p*_0_*I*_laser_/4. We assume *p*_0_ < 0, such that particles move towards low-intensity regions, and use an intensity profile corresponding to the potential (38). If, as assumed in many theoretical studies of such systems, the particles are passive and noninteracting, the density profile of the particles in case of a beam profile of the form (38) is given by^90^

54 $$\rho=\bar{\rho }\,\left(1-\frac{{U}_{1}-{\bar{U}}_{1}}{{k}_{{{{{{{{{{\rm{B}}}}}}}}}}}T}\right),$$

where ${\bar{U}}_{1}$ is the spatial average of *U*_1_. In our active interacting system, however, the discontinuous transition in Eq. (54) is replaced by a smoother one as shown in Fig. 2. Interacting active particles are thus more likely to be found in the illuminated region than passive particles. A visualization of this proposed experiment can be found in Fig. 3.

Next, we discuss what the active tunnel effect adds to the existing literature. First and most obviously, it constitutes an analytical solution to AMB/AMI (namely Eq. (47)) that is analogous to a quantum-mechanical one (namely Eq. (43)). Second, it shows how activity and interactions affect the interaction with an external potential, namely by smoothing the density profile at a sharp barrier (to see this, compare Eqs. (47) and (54)). Third, it offers, as in the case of FDM, a soft-matter-based physical intuition for where the tunnel effect comes from. Tunneling is only possible for *ℏ* > 0, and the terms proportional to *ℏ*^2^ (quantum potential) correspond to the gradient terms in the active matter model. Gradient terms penalize sharp interfaces and thus lead to a smooth transition of the density at a potential barrier. But the fact that the quantum-mechanical probability density decays smoothly on a finite length scale ϰ^−1^ inside the barrier is precisely what is characteristic of the tunnel effect in quantum mechanics. Hence, quantum tunneling can be thought of as arising from the energetic cost of density gradients.

Also, we should address the differences between the active and the quantum-mechanical tunnel effect. An important one is the different physical interpretation. The active tunnel effect is related to the density of classical particles, the quantum tunnel effect to the probability that a quantum-mechanical particle overcomes a potential barrier. Moreover, the active tunnel effect is more complex since it can be affected by a larger number of parameters (as illustrated in Fig. 2). For example, by considering the more general case ${f}^{{\prime} }(\rho ) \,\ne\, 0$, where AMI becomes an analog of the nonlinear Schrödinger equation (31) instead, we can consider also nonlinear (soliton) tunneling^91^, an effect that is of importance in optics^92^.

## Discussion

In this work, we have systematically derived an extension of common scalar active matter models to the underdamped case which we refer to as active model I+. This model and its derivation reveal some important properties of inertial active matter, such as the fact that mechanical and thermodynamic definitions of the velocity give different results and that the particles’ density-dependent swimming speed acts as an effective viscosity. Moreover, we have shown that AMI+ contains (a nonlinear extension of) the Madelung equations and therefore an analog of the (nonlinear) Schrödinger equation as a special case, such that the Schrödinger equation can be seen as an active field theory. This allows to study quantum effects in active-matter systems, as has been demonstrated for the tunnel effect and for fuzzy dark matter. A numerical investigation of the active tunnel effect shows that this active-quantum analogy has no sensitive dependence on the assumptions that have been made to derive it, indicating that it is of broader relevance for both theory and experiment.

## Methods

### Microscopic derivation of active model I+

Here, we explain the microscopic derivation of AMI+. A visualization of this derivation can be found in Fig. 1.

**Fig. 4:**
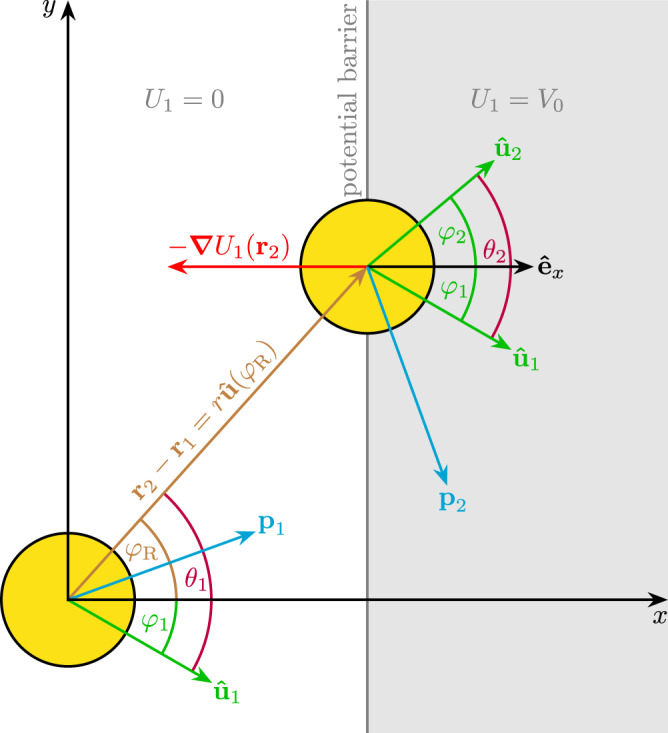
Visualization of the microscopic setup considered in the derivation. Here, *x* and *y* are the components of **r** and ${{{{\hat{{{{{{{\bf{e}}}}}}}}}}}}_{x}$ is the unit vector in *x* direction. Each particle’s state is characterized by the degrees of freedom **r**_*i*_ (position), **p**_*i*_ (momentum), and ${\hat{{{{{{{{{{\bf{u}}}}}}}}}}}}_{i}$ (orientation). By exploiting the symmetries of the system, we can pass to a reduced description in terms of the vector $r\hat{{{{{{{{{{\bf{u}}}}}}}}}}}({\varphi }_{{{{{{{{{{\rm{R}}}}}}}}}}})$ pointing from particle 1 to particle 2 and the angles *θ*_1_ and *θ*_2_ defined relative to the orientation of particle 1. A force arises at the boundary of the potential barrier inside of which *U*_1_ = *V*_0_. The setup is a generalization of the overdamped one shown in Fig. 1 of the article by Jeggle et al.^93^.

Microscopically, a two-dimensional system of *N* underdamped ABPs is described by the Langevin equations^35^

55 $${\dot{{{{{{{{{{\bf{r}}}}}}}}}}}}_{i}=\frac{{{{{{{{{{{\bf{p}}}}}}}}}}}_{i}}{m},$$

56 $${\dot{{{{{{{{{{\bf{p}}}}}}}}}}}}_{i}=-\gamma {{{{{{{{{{\bf{p}}}}}}}}}}}_{i}-{{{{{{{{{{\boldsymbol{\nabla }}}}}}}}}}}_{{{{{{{{{{{\bf{r}}}}}}}}}}}_{i}}U(\{{{{{{{{{{{\bf{r}}}}}}}}}}}_{i}\})+m\gamma {v}_{0}{\hat{{{{{{{{{{\bf{u}}}}}}}}}}}}_{i}+{{{{{{{{{{{\mathbf{\upeta }}}}}}}}}}}}_{i},$$

57 $${\dot{\varphi }}_{i}={\chi }_{i},$$

where **r**_*i*_(*t*), **p**_*i*_(*t*), and *φ*_*i*_(*t*) are position, momentum, and orientation (direction of self-propulsion force) of the *i*-th particle, ${\hat{{{{{{{{{{\bf{u}}}}}}}}}}}}_{i}({\varphi }_{i})={(\cos ({\varphi }_{i}),\sin ({\varphi }_{i}))}^{{{{{{{{{{\rm{T}}}}}}}}}}}$ is its orientation vector, *m* is its mass, *v*_0_ is its self-propulsion velocity, *γ* is the translational friction coefficient, and *U* = *U*_2_ + *U*_1_ is the potential consisting of interaction potential *U*_2_ and external potential *U*_1_. The translational noises **η**_*i*_ and the rotational noises *χ*_*i*_(*t*) have the properties

58 $$\langle {{{{{{{{{{\mathbf{\eta }}}}}}}}}}}_{i}(t)\rangle={{{{{{{{{\bf{0}}}}}}}}}},$$

59 $$\langle {{{{{{{{{{\mathbf{\eta }}}}}}}}}}}_{i}(t)\otimes {{{{{{{{{{\mathbf{\eta }}}}}}}}}}}_{j}({t}^{{\prime} })\rangle=2\gamma m{k}_{{{{{{{{{{\rm{B}}}}}}}}}}}T{\mathbb{1}}{\delta }_{ij}\delta (t-{t}^{{\prime} }),$$

60 $$\langle {\chi }_{i}(t)\rangle=0,$$

61 $$\langle {\chi }_{i}(t){\chi }_{j}({t}^{{\prime} })\rangle=2{D}_{{{{{{{{{{\rm{R}}}}}}}}}}}{\delta }_{ij}\delta (t-{t}^{{\prime} }),$$

with the ensemble average 〈⋅〉 and the unit matrix ${\mathbb{1}}$. This setup is visualized in Fig. 4, which shows the independent degrees of freedom of each particle (position, momentum, and orientation) and the forces from an external potential (which in the figure has the form of a barrier as in the active tunnel effect). The corresponding Fokker-Planck equation is given by^35^

62 $${\dot{P}}_{N}(\{{{{{{{{{{{\bf{r}}}}}}}}}}}_{i},{{{{{{{{{{\bf{p}}}}}}}}}}}_{i},{\hat{{{{{{{{{{\bf{u}}}}}}}}}}}}_{i}\})={{{{{{{{{\rm{i}}}}}}}}}}L(\{{{{{{{{{{{\bf{r}}}}}}}}}}}_{i},{{{{{{{{{{\bf{p}}}}}}}}}}}_{i},{\hat{{{{{{{{{{\bf{u}}}}}}}}}}}}_{i}\}){P}_{N}(\{{{{{{{{{{{\bf{r}}}}}}}}}}}_{i},{{{{{{{{{{\bf{p}}}}}}}}}}}_{i},{\hat{{{{{{{{{{\bf{u}}}}}}}}}}}}_{i}\}),$$

where *P*_*N*_ is the *N*-body probability distribution and

63 $${{{{{{{{{\rm{i}}}}}}}}}}L(\{{{{{{{{{{{\bf{r}}}}}}}}}}}_{i},{{{{{{{{{{\bf{p}}}}}}}}}}}_{i},{\hat{{{{{{{{{{\bf{u}}}}}}}}}}}}_{i}\})=	\mathop{\sum }\limits_{i=1}^{N}\,\left(-\frac{{{{{{{{{{{\bf{p}}}}}}}}}}}_{i}}{m}\cdot {{{{{{{{{{\boldsymbol{\nabla }}}}}}}}}}}_{{{{{{{{{{{\rm{r}}}}}}}}}}}_{i}}+\gamma+\gamma {{{{{{{{{{\bf{p}}}}}}}}}}}_{i}\cdot {{{{{{{{{{\boldsymbol{\nabla }}}}}}}}}}}_{{{{{{{{{{{\bf{p}}}}}}}}}}}_{i}}+({{{{{{{{{{\boldsymbol{\nabla }}}}}}}}}}}_{{{{{{{{{{{\bf{r}}}}}}}}}}}_{i}}U)\cdot {{{{{{{{{{\boldsymbol{\nabla }}}}}}}}}}}_{{{{{{{{{{{\bf{p}}}}}}}}}}}_{i}}\right. \\ 	\left.-\,m\gamma {v}_{0}{\hat{{{{{{{{{{\bf{u}}}}}}}}}}}}_{i}\cdot {{{{{{{{{{\boldsymbol{\nabla }}}}}}}}}}}_{{{{{{{{{{{\bf{p}}}}}}}}}}}_{i}}+\gamma m{k}_{{{{{{{{{{\rm{B}}}}}}}}}}}T{{{{{{{{{{\boldsymbol{\nabla }}}}}}}}}}}_{{{{{{{{{{{\bf{p}}}}}}}}}}}_{i}}^{2}+{D}_{{{{{{{{{{\rm{R}}}}}}}}}}}{\partial }_{{\varphi }_{i}}^{2}\right)$$

is the Liouvillian. The dependence on *t* is not written explicitly to simplify the notation (except in Eqs. (58)–(61), where it is important).

By integrating Eq. (62) over the coordinates of all except for one particle, we find^35^

64 $${\dot{P}}_{1}({{{{{{{{{\bf{r}}}}}}}}}},{{{{{{{{{\bf{p}}}}}}}}}},\hat{{{{{{{{{{\bf{u}}}}}}}}}}})=	\, \,\left(-\frac{{{{{{{{{{\bf{p}}}}}}}}}}}{m}\cdot {{{{{{{{{\boldsymbol{\nabla }}}}}}}}}}+\gamma+\gamma {{{{{{{{{\bf{p}}}}}}}}}}\cdot {{{{{{{{{{\boldsymbol{\nabla }}}}}}}}}}}_{{{{{{{{{{\bf{p}}}}}}}}}}}+({{{{{{{{{\boldsymbol{\nabla }}}}}}}}}}{U}_{1})\cdot {{{{{{{{{{\boldsymbol{\nabla }}}}}}}}}}}_{{{{{{{{{{\bf{p}}}}}}}}}}}\right.\\ 	-\,\left.m\gamma {v}_{0}\hat{{{{{{{{{{\bf{u}}}}}}}}}}}\cdot {{{{{{{{{{\boldsymbol{\nabla }}}}}}}}}}}_{{{{{{{{{{\bf{p}}}}}}}}}}}+\gamma m{k}_{{{{{{{{{{\rm{B}}}}}}}}}}}T{{{{{{{{{{\boldsymbol{\nabla }}}}}}}}}}}_{{{{{{{{{{\bf{p}}}}}}}}}}}^{2}+{D}_{{{{{{{{{{\rm{R}}}}}}}}}}}{\partial }_{\varphi }^{2}\right){P}_{1}({{{{{{{{{\bf{r}}}}}}}}}},{{{{{{{{{\bf{p}}}}}}}}}},\hat{{{{{{{{{{\bf{u}}}}}}}}}}})\\ 	+\int\,{{{{{{{{{{\rm{d}}}}}}}}}}}^{2}{r}_{2}\int\,{d}^{2}{p}_{2}\int\nolimits_{0}^{2\pi }{{{{{{{{{\rm{d}}}}}}}}}}{\varphi }_{2}({{{{{{{{{\boldsymbol{\nabla }}}}}}}}}}{U}_{2})\cdot {{{{{{{{{{\boldsymbol{\nabla }}}}}}}}}}}_{{{{{{{{{{\bf{p}}}}}}}}}}}{P}_{2}({{{{{{{{{\bf{r}}}}}}}}}},{{{{{{{{{{\bf{r}}}}}}}}}}}_{2},{{{{{{{{{\bf{p}}}}}}}}}},{{{{{{{{{{\bf{p}}}}}}}}}}}_{2},\hat{{{{{{{{{{\bf{u}}}}}}}}}}},{\hat{{{{{{{{{{\bf{u}}}}}}}}}}}}_{2})$$

with **∇** = **∇**_**r**_ and the *n*-body density defined as^58^

65 $${P}_{n}=	\frac{N!}{(N-n)!}\int\,{{{{{{{{{{\rm{d}}}}}}}}}}}^{2}{r}_{1}\cdots \int\,{{{{{{{{{{\rm{d}}}}}}}}}}}^{2}{r}_{N-n}\int\,{{{{{{{{{{\rm{d}}}}}}}}}}}^{2}{p}_{1}\cdots \int\,{{{{{{{{{{\rm{d}}}}}}}}}}}^{2}{p}_{N-n}\\ 	\int\nolimits_{0}^{2\pi }{{{{{{{{{\rm{d}}}}}}}}}}{\varphi }_{1}\cdots \int\nolimits_{0}^{2\pi }{{{{{{{{{\rm{d}}}}}}}}}}{\varphi }_{N-n}{P}_{N},$$

where *n* ∈ {1, …, *N*}. The index 1 is dropped for the coordinates. We define the particle density

66 $$\varrho ({{{{{{{{{\bf{r}}}}}}}}}},\hat{{{{{{{{{{\bf{u}}}}}}}}}}})=\int\,{{{{{{{{{{\rm{d}}}}}}}}}}}^{2}p{P}_{1}({{{{{{{{{\bf{r}}}}}}}}}},{{{{{{{{{\bf{p}}}}}}}}}},\hat{{{{{{{{{{\bf{u}}}}}}}}}}}).$$

Moreover, we make the generalized local equilibrium approximation (13) (see Results), which implies^35^

67 $$\varrho ({{{{{{{{{\bf{r}}}}}}}}}},\hat{{{{{{{{{{\bf{u}}}}}}}}}}}){\mathfrak{v}}({{{{{{{{{\bf{r}}}}}}}}}},\hat{{{{{{{{{{\bf{u}}}}}}}}}}})=\int\,{{{{{{{{{{\rm{d}}}}}}}}}}}^{2}p\frac{{{{{{{{{{\bf{p}}}}}}}}}}}{m}{P}_{1}({{{{{{{{{\bf{r}}}}}}}}}},{{{{{{{{{\bf{p}}}}}}}}}},\hat{{{{{{{{{{\bf{u}}}}}}}}}}}).$$

We now drop arguments of the fields unless unclear. Integrating Eq. (64) over **p** and using Eqs. (66) and (67) yields

68 $$\dot{\varrho }=-{{{{{{{{{\boldsymbol{\nabla }}}}}}}}}}\cdot (\varrho {\mathfrak{v}})+{D}_{{{{{{{{{{\rm{R}}}}}}}}}}}{\partial }_{\varphi }^{2}\varrho .$$

Similarly, we can multiply Eq. (64) by **p**/*m*, integrate over **p**, and use Eqs. (13) and (66)–(68) to get

69 $$\dot{{\mathfrak{v}}}+({\mathfrak{v}}\cdot {{{{{{{{{\boldsymbol{\nabla }}}}}}}}}}){\mathfrak{v}}=	-{D}_{{{{{{{{{{\rm{R}}}}}}}}}}}{\mathfrak{v}}\frac{{\partial }_{\varphi }^{2}\varrho }{\varrho }-\gamma {\mathfrak{v}}+\gamma {v}_{0}\hat{{{{{{{{{{\bf{u}}}}}}}}}}}-\frac{1}{m}{{{{{{{{{\boldsymbol{\nabla }}}}}}}}}}{U}_{1}\\ 	-\frac{{k}_{{{{{{{{{{\rm{B}}}}}}}}}}}T}{m}{{{{{{{{{\boldsymbol{\nabla }}}}}}}}}}\ln (\varrho )-\frac{1}{m\varrho }{{{{{{{{{\mathcal{I}}}}}}}}}}$$

with the interaction term

70 $${{{{{{{{{\mathcal{I}}}}}}}}}}({{{{{{{{{\bf{r}}}}}}}}}},\hat{{{{{{{{{{\bf{u}}}}}}}}}}})=\int\,{{{{{{{{{{\rm{d}}}}}}}}}}}^{2}{r}_{2}\int\nolimits_{0}^{2\pi }{{{{{{{{{\rm{d}}}}}}}}}}{\varphi }_{2}{\varrho }_{2}({{{{{{{{{\bf{r}}}}}}}}}},{{{{{{{{{{\bf{r}}}}}}}}}}}_{2},\hat{{{{{{{{{{\bf{u}}}}}}}}}}},{\hat{{{{{{{{{{\bf{u}}}}}}}}}}}}_{2}){{{{{{{{{\boldsymbol{\nabla }}}}}}}}}}{U}_{2}(r),$$

where *ϱ*_2_ = ∫ d^2^*p*∫ d^2^*p*_2_*P*_2_ is the two-particle density and *r* = ∥**r** − **r**_2_∥ with the Euclidean norm ∥⋅∥ is a distance. (From now on, we ignore factors of Λ^2^ or *ρ*_0_ required to make the argument of the logarithm dimensionless.) The derivation of Eq. (69) generally follows the standard procedure of deriving hydrodynamic equations from microscopic dynamics^58^. Our result differs from the standard form of velocity transport equations by the presence of the term $-{D}_{{{{{{{{{{\rm{R}}}}}}}}}}}{\mathfrak{v}}({\partial }_{\varphi }^{2}\varrho )/\varrho$, which arises from the term ${D}_{{{{{{{{{{\rm{R}}}}}}}}}}}{\partial }_{\varphi }^{2}\varrho$ in Eq. (68).

We can define the pair-distribution function *g* as^46,49,93^

71 $$g({{{{{{{{{\bf{r}}}}}}}}}},{{{{{{{{{{\bf{r}}}}}}}}}}}_{2},\hat{{{{{{{{{{\bf{u}}}}}}}}}}},{\hat{{{{{{{{{{\bf{u}}}}}}}}}}}}_{2})=\frac{{\varrho }_{2}({{{{{{{{{\bf{r}}}}}}}}}},{{{{{{{{{{\bf{r}}}}}}}}}}}_{2},\hat{{{{{{{{{{\bf{u}}}}}}}}}}},{\hat{{{{{{{{{{\bf{u}}}}}}}}}}}}_{2})}{\varrho ({{{{{{{{{\bf{r}}}}}}}}}},\hat{{{{{{{{{{\bf{u}}}}}}}}}}})\varrho ({{{{{{{{{{\bf{r}}}}}}}}}}}_{2},{\hat{{{{{{{{{{\bf{u}}}}}}}}}}}}_{2})}.$$

Following the treatment by Bickmann and Wittkowski^49^, we assume the pair-distribution function to be translationally and rotationally invariant, implying that it can be written as *g*(*r*, *θ*_1_, *θ*_2_) with the angles *θ*_1_ = *φ*_R_ − *φ* and *θ*_2_ = *φ*_2_ − *φ* and the parametrization ${{{{{{{{{{\bf{r}}}}}}}}}}}_{2}-{{{{{{{{{\bf{r}}}}}}}}}}=r\hat{{{{{{{{{{\bf{u}}}}}}}}}}}({\varphi }_{{{{{{{{{{\rm{R}}}}}}}}}}})$. These new variables are visualized in Fig. 4. Then, we can perform a Fourier and a gradient expansion^49,94^ of *g* and find

72 $${{{{{{{{{\mathcal{I}}}}}}}}}}({{{{{{{{{\bf{r}}}}}}}}}},\hat{{{{{{{{{{\bf{u}}}}}}}}}}})	=-\mathop{\sum }\limits_{l=0}^{\infty }\frac{1}{l!}\varrho ({{{{{{{{{\bf{r}}}}}}}}}},\varphi )\int\nolimits_{0}^{\infty }{{{{{{{{{\rm{d}}}}}}}}}}r{r}^{l+1}{U}_{2}^{{\prime} }(r)\int\nolimits_{0}^{2\pi }{{{{{{{{{\rm{d}}}}}}}}}}{\varphi }_{{{{{{{{{{\rm{R}}}}}}}}}}}\hat{{{{{{{{{{\bf{u}}}}}}}}}}}({\varphi }_{{{{{{{{{{\rm{R}}}}}}}}}}}){(\hat{{{{{{{{{{\bf{u}}}}}}}}}}}({\varphi }_{{{{{{{{{{\rm{R}}}}}}}}}}})\cdot {{{{{{{{{\boldsymbol{\nabla }}}}}}}}}})}^{l}\\ 	 \int\nolimits_{0}^{2\pi }{{{{{{{{{\rm{d}}}}}}}}}}{\varphi }_{2}\mathop{\sum }\limits_{{n}_{1},{n}_{2}=-\infty }^{\infty }{g}_{{n}_{1}{n}_{2}}(r)\cos ({n}_{1}{\theta }_{1}+{n}_{2}{\theta }_{2})\varrho ({{{{{{{{{\bf{r}}}}}}}}}},{\varphi }_{2})$$

with the *r*-dependent expansion coefficients^49^

73 $${g}_{{n}_{1}{n}_{2}}(r)=\frac{\int\nolimits_{0}^{2\pi }{{{{{{{{{\rm{d}}}}}}}}}}{\theta }_{1}\int\nolimits_{0}^{2\pi }{{{{{{{{{\rm{d}}}}}}}}}}{\theta }_{2}\,g(r,{\theta }_{1},{\theta }_{2})\cos ({n}_{1}{\theta }_{1}+{n}_{2}{\theta }_{2})}{{\pi }^{2}(1+{\delta }_{{n}_{1}0})(1+{\delta }_{{n}_{2}0})}$$

and $${U}_{2}^{{\prime} }(r)={{{{{{{{{\rm{d}}}}}}}}}}{U}_{2}/{{{{{{{{{\rm{d}}}}}}}}}}r$$.

We now carry out the Cartesian orientational expansions^7^

74 $$\varrho ({{{{{{{{{\bf{r}}}}}}}}}},\hat{{{{{{{{{{\bf{u}}}}}}}}}}})=\rho ({{{{{{{{{\bf{r}}}}}}}}}})+\hat{{{{{{{{{{\bf{u}}}}}}}}}}}\cdot {{{{{{{{{\bf{P}}}}}}}}}}({{{{{{{{{\bf{r}}}}}}}}}}),$$

75 $${{\mathfrak{v}}}({{{\bf{r}}}}, \hat{{{\bf{u}}}})={{{\bf{v}}}}({{{\bf{r}}}})+ \hat{{{\bf{u}}}} \cdot {\underline{{{\boldsymbol{ v}}}}}_{{{\bf{P}}}}({{{\bf{r}}}})$$

with the non-orientational particle density

76 $$\rho ({{{{{{{{{\bf{r}}}}}}}}}})=\frac{1}{2\pi }\int\nolimits_{0}^{2\pi }{{{{{{{{{\rm{d}}}}}}}}}}\varphi \varrho ({{{{{{{{{\bf{r}}}}}}}}}},\hat{{{{{{{{{{\bf{u}}}}}}}}}}}),$$

the local velocity

77 $${{{{{{{{{\bf{v}}}}}}}}}}({{{{{{{{{\bf{r}}}}}}}}}})=\frac{1}{2\pi }\int\nolimits_{0}^{2\pi }{{{{{{{{{\rm{d}}}}}}}}}}\varphi {\mathfrak{v}}({{{{{{{{{\bf{r}}}}}}}}}},\hat{{{{{{{{{{\bf{u}}}}}}}}}}}),$$

the local polarization

78 $${{{{{{{{{\bf{P}}}}}}}}}}({{{{{{{{{\bf{r}}}}}}}}}})=\frac{1}{\pi }\int\nolimits_{0}^{2\pi }{{{{{{{{{\rm{d}}}}}}}}}}\varphi \hat{{{{{{{{{{\bf{u}}}}}}}}}}}\varrho ({{{{{{{{{\bf{r}}}}}}}}}},\hat{{{{{{{{{{\bf{u}}}}}}}}}}}),$$

and the local velocity polarization

79 $${\underline{{{{{{{{{{\boldsymbol{v}}}}}}}}}}}}_{{{{{{{{{{\bf{P}}}}}}}}}}}({{{{{{{{{\bf{r}}}}}}}}}})=\frac{1}{\pi }\int\nolimits_{0}^{2\pi }{{{{{{{{{\rm{d}}}}}}}}}}\varphi \hat{{{{{{{{{{\bf{u}}}}}}}}}}}\otimes {\mathfrak{v}}({{{{{{{{{\bf{r}}}}}}}}}},\hat{{{{{{{{{{\bf{u}}}}}}}}}}}).$$

Here, our treatment differs in an important way from standard treatments of active overdamped^48,49^, passive underdamped^58^, and even active underdamped^35^ particles. Since we have a generalized velocity field ${\mathfrak{v}}$ that also depends on $\hat{{{{{{{{{{\bf{u}}}}}}}}}}}$, we have to perform the orientational expansion not only for the density, but also for the velocity.

We now insert Eq. (74) into $\ln (\varrho )$ and Taylor expand around **P** = **0**. This gives

80 $$\ln (\varrho ) \,\approx\, \ln (\rho )+\frac{1}{\rho }\hat{{{{{{{{{{\bf{u}}}}}}}}}}}\cdot {{{{{{{{{\bf{P}}}}}}}}}} \,\approx\, \ln (\rho )+\frac{1}{{\varrho }_{0}}\hat{{{{{{{{{{\bf{u}}}}}}}}}}}\cdot {{{{{{{{{\bf{P}}}}}}}}}},$$

where we have replaced *ρ* by a spatially and temporally constant reference density *ϱ*_0_ in the last step. Similarly, we insert Eqs. (74) and (75) into ${\mathfrak{v}}({\partial }_{\varphi }^{2}\varrho )/\varrho$ and Taylor expand around **P** = **0** to find

81 $$\frac{({{{{{{{{{\bf{v}}}}}}}}}}+\hat{{{{{{{{{{\bf{u}}}}}}}}}}}\cdot {\underline{{{{{{{{{{\boldsymbol{v}}}}}}}}}}}}_{{{{{{{{{{\rm{P}}}}}}}}}}}){\partial }_{\varphi }^{2}(\rho+\hat{{{{{{{{{{\bf{u}}}}}}}}}}}\cdot {{{{{{{{{\bf{P}}}}}}}}}})}{\rho+\hat{{{{{{{{{{\bf{u}}}}}}}}}}}\cdot {{{{{{{{{\bf{P}}}}}}}}}}} \,\approx\, -\frac{({{{{{{{{{\bf{v}}}}}}}}}}+\hat{{{{{{{{{{\bf{u}}}}}}}}}}}\cdot {\underline{{{{{{{{{{\boldsymbol{v}}}}}}}}}}}}_{{{{{{{{{{\rm{P}}}}}}}}}}})\hat{{{{{{{{{{\bf{u}}}}}}}}}}}\cdot {{{{{{{{{\bf{P}}}}}}}}}}}{\rho }.$$

Finally, an orientational expansion of the interaction term gives

82 $$\frac{1}{\varrho }{{{{{{{{{\mathcal{I}}}}}}}}}}={A}_{1}\hat{{{{{{{{{{\bf{u}}}}}}}}}}}\rho+{A}_{2}{{{{{{{{{\boldsymbol{\nabla }}}}}}}}}}\rho+{A}_{3}{{{{{{{{{{\boldsymbol{\nabla }}}}}}}}}}}^{2}{{{{{{{{{\bf{P}}}}}}}}}}+2{A}_{3}{{{{{{{{{\boldsymbol{\nabla }}}}}}}}}}({{{{{{{{{\boldsymbol{\nabla }}}}}}}}}}\cdot {{{{{{{{{\bf{P}}}}}}}}}})\\+{A}_{4}\hat{{{{{{{{{{\bf{u}}}}}}}}}}}{{{{{{{{{{\boldsymbol{\nabla }}}}}}}}}}}^{2}\rho+2{A}_{4}{{{{{{{{{\boldsymbol{\nabla }}}}}}}}}}({{{{{{{{{\boldsymbol{\nabla }}}}}}}}}}\cdot \hat{{{{{{{{{{\bf{u}}}}}}}}}}})\rho+\cdots$$

with the coefficients

83 $${A}_{1}=-2{\pi }^{2}\int\nolimits_{0}^{\infty }{{{{{{{{{\rm{d}}}}}}}}}}rr{U}_{2}^{{\prime} }(r)({g}_{1,0}(r)+{g}_{-1,0}(r)),$$

84 $${A}_{2}=-2{\pi }^{2}\int\nolimits_{0}^{\infty }{{{{{{{{{\rm{d}}}}}}}}}}r{r}^{2}{U}_{2}^{{\prime} }(r){g}_{0,0}(r),$$

85 $${A}_{3}=-\frac{{\pi }^{2}}{4}\int\nolimits_{0}^{\infty }{{{{{{{{{\rm{d}}}}}}}}}}r{r}^{3}{U}_{2}^{{\prime} }(r)({g}_{1,-1}(r)+{g}_{-1,1}(r)),$$

86 $${A}_{4}=-\frac{{\pi }^{2}}{2}\int\nolimits_{0}^{\infty }{{{{{{{{{\rm{d}}}}}}}}}}r{r}^{3}{U}_{2}^{{\prime} }(r)({g}_{1,0}(r)+{g}_{-1,0}(r)).$$

These coefficients can be time-dependent by inheriting a time-dependence of *g*^49^, but we will assume them to be constant.

From Eqs. (68), (69), (74), (75), and (80)–(82), we obtain the general local field theory for underdamped ABPs

87 $$\dot{\rho }=-{{{{{{{{{\boldsymbol{\nabla }}}}}}}}}}\cdot (\rho {{{{{{{{{\bf{v}}}}}}}}}})-\frac{1}{2}{{{{{{{{{\boldsymbol{\nabla }}}}}}}}}}\cdot ({{{{{{{{{\bf{P}}}}}}}}}}\cdot {\underline{{{{{{{{{{\boldsymbol{v}}}}}}}}}}}}_{{{{{{{{{{\bf{P}}}}}}}}}}}),$$

88 $$\dot{{{{{{{{{{\bf{P}}}}}}}}}}}=-{{{{{{{{{\boldsymbol{\nabla }}}}}}}}}}\cdot ({{{{{{{{{\bf{v}}}}}}}}}}\otimes {{{{{{{{{\bf{P}}}}}}}}}})-{{{{{{{{{\boldsymbol{\nabla }}}}}}}}}}\cdot (\rho {\underline{{{{{{{{{{\boldsymbol{v}}}}}}}}}}}}_{{{{{{{{{{\bf{P}}}}}}}}}}})-{D}_{{{{{{{{{{\rm{R}}}}}}}}}}}{{{{{{{{{\bf{P}}}}}}}}}},$$

89 $$\dot{{{{{{{{{{\bf{v}}}}}}}}}}}=	-({{{{{{{{{\bf{v}}}}}}}}}}\cdot {{{{{{{{{\boldsymbol{\nabla }}}}}}}}}}){{{{{{{{{\bf{v}}}}}}}}}}-\frac{1}{2}({\underline{{{{{{{{{{\boldsymbol{v}}}}}}}}}}}}_{{{{{{{{{{\bf{P}}}}}}}}}}}\cdot {{{{{{{{{\boldsymbol{\nabla }}}}}}}}}})\cdot {\underline{{{{{{{{{{\boldsymbol{v}}}}}}}}}}}}_{{{{{{{{{{\bf{P}}}}}}}}}}}-\gamma {{{{{{{{{\bf{v}}}}}}}}}}-\frac{{A}_{3}}{m}{{{{{{{{{{\boldsymbol{\nabla }}}}}}}}}}}^{2}{{{{{{{{{\bf{P}}}}}}}}}}\\ 	+{D}_{{{{{{{{{{\rm{R}}}}}}}}}}}\frac{{{{{{{{{{\bf{P}}}}}}}}}}\cdot {\underline{{{{{{{{{{\boldsymbol{v}}}}}}}}}}}}_{{{{{{{{{{\bf{P}}}}}}}}}}}}{2\rho }-\frac{1}{m}{{{{{{{{{\boldsymbol{\nabla }}}}}}}}}}\,\left({k}_{{{{{{{{{{\rm{B}}}}}}}}}}}T\ln (\rho )+{A}_{2}\rho+\,2{A}_{3}({{{{{{{{{\boldsymbol{\nabla }}}}}}}}}}\cdot {{{{{{{{{\bf{P}}}}}}}}}})+{U}_{1}\right),$$

90 $${\underline{{{{\dot{{{{{{{\boldsymbol{v}}}}}}}}}}}}}_{{{{{{{{{{\bf{P}}}}}}}}}}}=	-({\underline{{{{{{{{{{\boldsymbol{v}}}}}}}}}}}}_{{{{{{{{{{\rm{P}}}}}}}}}}}\cdot {{{{{{{{{\boldsymbol{\nabla }}}}}}}}}})\otimes {{{{{{{{{\bf{v}}}}}}}}}}-({{{{{{{{{\bf{v}}}}}}}}}}\cdot {{{{{{{{{\boldsymbol{\nabla }}}}}}}}}}){\underline{{{{{{{{{{\boldsymbol{v}}}}}}}}}}}}_{{{{{{{{{{\bf{P}}}}}}}}}}}-\gamma {\underline{{{{{{{{{{\boldsymbol{v}}}}}}}}}}}}_{{{{{{{{{{\bf{P}}}}}}}}}}}+\gamma {v}_{0}{\mathbb{1}}\\ 	+{D}_{{{{{{{{{{\rm{R}}}}}}}}}}}\frac{{{{{{{{{{\bf{v}}}}}}}}}}\otimes {{{{{{{{{\bf{P}}}}}}}}}}}{\rho }-\frac{{k}_{{{{{{{{{{\rm{B}}}}}}}}}}}T}{{\varrho }_{0}m}{{{{{{{{{\boldsymbol{\nabla }}}}}}}}}}\otimes {{{{{{{{{\bf{P}}}}}}}}}}-\frac{{\mathbb{1}}}{m}({A}_{1}\rho+{A}_{4}{{{{{{{{{{\boldsymbol{\nabla }}}}}}}}}}}^{2}\rho )-\frac{2{A}_{4}}{m}{{{{{{{{{\boldsymbol{\nabla }}}}}}}}}}\otimes {{{{{{{{{\boldsymbol{\nabla }}}}}}}}}}\rho .$$

Starting from the very general model given by Eqs. (87)–(90), various approximations can be made. In most active matter models, it is assumed that the polarization **P** is slow compared to the velocity **v**. While this is reasonable for strongly damped systems, **v** should be slow in a system with weak damping and activity because there the momentum density is (almost) a conserved quantity (unlike **P**). In this case, it is plausible to assume that **v** evolves slower than **P**. This limit, which is less well understood, will be considered in this work.

Using the quasi-stationary approximation

91 $${\underline{{{{\dot{{{{{{{\boldsymbol{v}}}}}}}}}}}}}_{{{{{{{{{{\bf{P}}}}}}}}}}}=\underline{{{{{{{{{{\boldsymbol{0}}}}}}}}}}},$$

Eq. (90) gives

92 $${\underline{{{{{{{{{{\boldsymbol{v}}}}}}}}}}}}_{{{{{{{{{{\bf{P}}}}}}}}}}}=\,\left({v}_{{{{{{{{{{\rm{ld}}}}}}}}}}}(\rho )-\frac{{A}_{4}}{\gamma m}{{{{{{{{{{\boldsymbol{\nabla }}}}}}}}}}}^{2}\rho \right){\mathbb{1}}+{D}_{{{{{{{{{{\rm{R}}}}}}}}}}}\frac{{{{{{{{{{\bf{v}}}}}}}}}}\otimes {{{{{{{{{\bf{P}}}}}}}}}}}{\gamma \rho }\\ -\frac{2{A}_{4}}{\gamma m}{{{{{{{{{\boldsymbol{\nabla }}}}}}}}}}\otimes {{{{{{{{{\boldsymbol{\nabla }}}}}}}}}}\rho -\frac{{k}_{{{{{{{{{{\rm{B}}}}}}}}}}}T}{\gamma {\varrho }_{0}m}{{{{{{{{{\boldsymbol{\nabla }}}}}}}}}}\otimes {{{{{{{{{\bf{P}}}}}}}}}}\\ -\frac{1}{\gamma }({\underline{{{{{{{{{{\boldsymbol{v}}}}}}}}}}}}_{{{{{{{{{{\bf{P}}}}}}}}}}}\cdot {{{{{{{{{\boldsymbol{\nabla }}}}}}}}}})\otimes {{{{{{{{{\bf{v}}}}}}}}}}-\frac{1}{\gamma }({{{{{{{{{\bf{v}}}}}}}}}}\cdot {{{{{{{{{\boldsymbol{\nabla }}}}}}}}}}){\underline{{{{{{{{{{\boldsymbol{v}}}}}}}}}}}}_{{{{{{{{{{\bf{P}}}}}}}}}}},$$

where *v*_ld_ is defined in Eq. (4). By inserting Eq. (92) recursively into itself and neglecting terms of higher than second order in gradients, of higher than first order in velocities, or that involve products of polarizations with velocities, we find

93 $${\underline{{{{{{{{{{\boldsymbol{v}}}}}}}}}}}}_{{{{{{{{{{\bf{P}}}}}}}}}}}=	\,\left({v}_{{{{{{{{{{\rm{ld}}}}}}}}}}}(\rho )-\frac{{A}_{4}}{\gamma m}{{{{{{{{{{\boldsymbol{\nabla }}}}}}}}}}}^{2}\rho \right){\mathbb{1}}-\frac{2{A}_{4}}{\gamma m}{{{{{{{{{\boldsymbol{\nabla }}}}}}}}}}\otimes {{{{{{{{{\boldsymbol{\nabla }}}}}}}}}}\rho \\ 	-\frac{{k}_{{{{{{{{{{\rm{B}}}}}}}}}}}T}{\gamma {\varrho }_{0}m}{{{{{{{{{\boldsymbol{\nabla }}}}}}}}}}\otimes {{{{{{{{{\bf{P}}}}}}}}}}-\frac{{v}_{{{{{{{{{{\rm{ld}}}}}}}}}}}(\rho )}{\gamma }{{{{{{{{{\boldsymbol{\nabla }}}}}}}}}}\otimes {{{{{{{{{\bf{v}}}}}}}}}}+\frac{{A}_{1}}{{\gamma }^{2}m}({{{{{{{{{\bf{v}}}}}}}}}}\cdot {{{{{{{{{\boldsymbol{\nabla }}}}}}}}}})\rho {\mathbb{1}}.$$

The motivation behind these approximations is that we wish to derive a theory of third order in gradients and of second order in velocities and that we assume both polarizations and velocities to be small. (By velocity, we mean **v**, whereas ${\underline{{{{{{{{{{\boldsymbol{v}}}}}}}}}}}}_{{{{{{{{{{\bf{P}}}}}}}}}}}$ is always referred to as velocity polarization.) The velocity polarization ${\underline{{{{{{{{{{\boldsymbol{v}}}}}}}}}}}}_{{{{{{{{{{\bf{P}}}}}}}}}}}$ appears in Eq. (89) only in the term $({\underline{{{{{{{{{{\boldsymbol{v}}}}}}}}}}}}_{{{{{{{{{{\bf{P}}}}}}}}}}}\cdot {{{{{{{{{\boldsymbol{\nabla }}}}}}}}}})\cdot {\underline{{{{{{{{{{\boldsymbol{v}}}}}}}}}}}}_{{{{{{{{{{\bf{P}}}}}}}}}}}/2$ (quadratic in ${\underline{{{{{{{{{{\boldsymbol{v}}}}}}}}}}}}_{{{{{{{{{{\bf{P}}}}}}}}}}}$ and of first order in gradients) and in the term ${D}_{{{{{{{{{{\rm{R}}}}}}}}}}}{{{{{{{{{\bf{P}}}}}}}}}}\cdot {\underline{{{{{{{{{{\boldsymbol{v}}}}}}}}}}}}_{{{{{{{{{{\bf{P}}}}}}}}}}}/(2\rho )$ (product with the small polarization). If we insert Eq. (93) into Eq. (88) and drop again terms containing products of velocities with polarizations (in particular the advection term), we find

94 $$\dot{{{{{{{{{{\bf{P}}}}}}}}}}}=	-{{{{{{{{{\boldsymbol{\nabla }}}}}}}}}}(\rho {v}_{{{{{{{{{{\rm{ld}}}}}}}}}}}(\rho ))+\frac{{A}_{4}}{\gamma m}\,\left({{{{{{{{{\boldsymbol{\nabla }}}}}}}}}}{({{{{{{{{{\boldsymbol{\nabla }}}}}}}}}}\rho )}^{2}+3\rho {{{{{{{{{\boldsymbol{\nabla }}}}}}}}}}{{{{{{{{{{\boldsymbol{\nabla }}}}}}}}}}}^{2}\rho+({{{{{{{{{\boldsymbol{\nabla }}}}}}}}}}\rho )({{{{{{{{{{\boldsymbol{\nabla }}}}}}}}}}}^{2}\rho )\right)\\ 	+\frac{{k}_{{{{{{{{{{\rm{B}}}}}}}}}}}T}{\gamma {\varrho }_{0}m}{{{{{{{{{\boldsymbol{\nabla }}}}}}}}}}\cdot (\rho {{{{{{{{{\boldsymbol{\nabla }}}}}}}}}}\otimes {{{{{{{{{\bf{P}}}}}}}}}})+{{{{{{{{{\boldsymbol{\nabla }}}}}}}}}}\cdot \,\left(\frac{{v}_{{{{{{{{{{\rm{ld}}}}}}}}}}}(\rho )}{\gamma }\rho {{{{{{{{{\boldsymbol{\nabla }}}}}}}}}}\otimes {{{{{{{{{\bf{v}}}}}}}}}}\right)\\ 	-\frac{{A}_{1}}{{\gamma }^{2}m}{{{{{{{{{\boldsymbol{\nabla }}}}}}}}}}(\rho (({{{{{{{{{\bf{v}}}}}}}}}}\cdot {{{{{{{{{\boldsymbol{\nabla }}}}}}}}}})\rho ))-{D}_{{{{{{{{{{\rm{R}}}}}}}}}}}{{{{{{{{{\bf{P}}}}}}}}}},$$

where we used the vector identity

95 $${{{{{{{{{\boldsymbol{\nabla }}}}}}}}}}\cdot (\rho {{{{{{{{{\boldsymbol{\nabla }}}}}}}}}}\otimes {{{{{{{{{\boldsymbol{\nabla }}}}}}}}}}\rho )=\frac{1}{2}{{{{{{{{{\boldsymbol{\nabla }}}}}}}}}}{({{{{{{{{{\boldsymbol{\nabla }}}}}}}}}}\rho )}^{2}+\rho {{{{{{{{{\boldsymbol{\nabla }}}}}}}}}}{{{{{{{{{{\boldsymbol{\nabla }}}}}}}}}}}^{2}\rho .$$

If we have *v*_ld_(*ρ*) ≈ *v*_0_, the first term on the right-hand side of Eq. (94) reduces to the self-propulsion term known from the active PFC model^37^. We now make the further quasi-stationary approximation^46,48,49,95^

96 $$\dot{{{{{{{{{{\bf{P}}}}}}}}}}}={{{{{{{{{\bf{0}}}}}}}}}}$$

and find

97 $${{{{{{{{{\bf{P}}}}}}}}}}=	-\frac{1}{{D}_{{{{{{{{{{\rm{R}}}}}}}}}}}}{{{{{{{{{\boldsymbol{\nabla }}}}}}}}}}(\rho {v}_{{{{{{{{{{\rm{ld}}}}}}}}}}}(\rho ))+{{{{{{{{{\boldsymbol{\nabla }}}}}}}}}}\cdot \,\left(\frac{{v}_{{{{{{{{{{\rm{ld}}}}}}}}}}}(\rho )}{\gamma {D}_{{{{{{{{{{\rm{R}}}}}}}}}}}}\rho {{{{{{{{{\boldsymbol{\nabla }}}}}}}}}}\otimes {{{{{{{{{\bf{v}}}}}}}}}}\right)\\ 	+\frac{{A}_{4}}{\gamma m{D}_{{{{{{{{{{\rm{R}}}}}}}}}}}}\,\left({{{{{{{{{\boldsymbol{\nabla }}}}}}}}}}{({{{{{{{{{\boldsymbol{\nabla }}}}}}}}}}\rho )}^{2}+3\rho {{{{{{{{{\boldsymbol{\nabla }}}}}}}}}}{{{{{{{{{{\boldsymbol{\nabla }}}}}}}}}}}^{2}\rho+({{{{{{{{{\boldsymbol{\nabla }}}}}}}}}}\rho )({{{{{{{{{{\boldsymbol{\nabla }}}}}}}}}}}^{2}\rho )\right)\\ 	+\frac{{k}_{{{{{{{{{{\rm{B}}}}}}}}}}}T}{\gamma {\varrho }_{0}m{D}_{{{{{{{{{{\rm{R}}}}}}}}}}}}{{{{{{{{{\boldsymbol{\nabla }}}}}}}}}}\cdot (\rho {{{{{{{{{\boldsymbol{\nabla }}}}}}}}}}\otimes {{{{{{{{{\bf{P}}}}}}}}}})-\frac{{A}_{1}}{{\gamma }^{2}m{D}_{{{{{{{{{{\rm{R}}}}}}}}}}}}{{{{{{{{{\boldsymbol{\nabla }}}}}}}}}}(\rho (({{{{{{{{{\bf{v}}}}}}}}}}\cdot {{{{{{{{{\boldsymbol{\nabla }}}}}}}}}})\rho )).$$

Inserting Eq. (97) into itself and neglecting terms of higher than third order in gradients and second order in densities gives

98 $${{{{{{{\bf{P}}}}}}}}=	-\frac{1}{{D}_{{{{{{{{\rm{R}}}}}}}}}}{{{{{{{{{\boldsymbol{\nabla }}}}}}}}}}(\rho {v}_{{{{{{{{{{\rm{ld}}}}}}}}}}}(\rho ))+{{{{{{{{{\boldsymbol{\nabla }}}}}}}}}}\cdot \,\left(\frac{{v}_{{{{{{{{{{\rm{ld}}}}}}}}}}}(\rho )}{\gamma {D}_{{{{{{{{{{\rm{R}}}}}}}}}}}}\rho {{{{{{{{{\boldsymbol{\nabla }}}}}}}}}}\otimes {{{{{{{{{\bf{v}}}}}}}}}}\right)\\ 	+\frac{{A}_{4}}{\gamma m{D}_{{{{{{{{{{\rm{R}}}}}}}}}}}}\,\left({{{{{{{{{\boldsymbol{\nabla }}}}}}}}}}{({{{{{{{{{\boldsymbol{\nabla }}}}}}}}}}\rho )}^{2}+3\rho {{{{{{{{{\boldsymbol{\nabla }}}}}}}}}}{{{{{{{{{{\boldsymbol{\nabla }}}}}}}}}}}^{2}\rho+({{{{{{{{{\boldsymbol{\nabla }}}}}}}}}}\rho )({{{{{{{{{{\boldsymbol{\nabla }}}}}}}}}}}^{2}\rho )\right)\\ 	 -\frac{{v}_{0}{k}_{{{{{{{{{{\rm{B}}}}}}}}}}}T}{2\gamma {\varrho }_{0}m{D}_{{{{{{{{{{\rm{R}}}}}}}}}}}^{2}}({{{{{{{{{\boldsymbol{\nabla }}}}}}}}}}{({{{{{{{{{\boldsymbol{\nabla }}}}}}}}}}\rho )}^{2}+2\rho {{{{{{{{{\boldsymbol{\nabla }}}}}}}}}}{{{{{{{{{{\boldsymbol{\nabla }}}}}}}}}}}^{2}\rho )\\ 	 -\frac{{A}_{1}}{{\gamma }^{2}m{D}_{{{{{{{{{{\rm{R}}}}}}}}}}}}{{{{{{{{{\boldsymbol{\nabla }}}}}}}}}}(\rho (({{{{{{{{{\bf{v}}}}}}}}}}\cdot {{{{{{{{{\boldsymbol{\nabla }}}}}}}}}})\rho )),$$

where we have used Eq. (95) again. This agrees with the result from Bialké et al.^96^ if we neglect terms of higher order in gradients and the velocity term in Eq. (98). We can insert Eq. (98) into Eq. (93) and neglect terms of higher than third order in gradients to get

99 $${\underline{{{{{{{{{{\boldsymbol{v}}}}}}}}}}}}_{{{{{{{{{{\bf{P}}}}}}}}}}}=	{v}_{{{{{{{{{{\rm{D}}}}}}}}}}}[\rho,{{{{{{{{{\bf{v}}}}}}}}}}]{\mathbb{1}}-\,\left(\frac{2{A}_{4}}{\gamma m}-\frac{{v}_{0}{k}_{{{{{{{{{{\rm{B}}}}}}}}}}}T}{\gamma {\varrho }_{0}m{D}_{{{{{{{{{{\rm{R}}}}}}}}}}}}\right){{{{{{{{{\boldsymbol{\nabla }}}}}}}}}}\otimes {{{{{{{{{\boldsymbol{\nabla }}}}}}}}}}\rho \\ 	 -\frac{{k}_{{{{{{{{{{\rm{B}}}}}}}}}}}T{A}_{1}}{{\gamma }^{2}{\varrho }_{0}{m}^{2}{D}_{{{{{{{{{{\rm{R}}}}}}}}}}}}{{{{{{{{{\boldsymbol{\nabla }}}}}}}}}}\otimes {{{{{{{{{\boldsymbol{\nabla }}}}}}}}}}{\rho }^{2}-\frac{{v}_{{{{{{{{{{\rm{ld}}}}}}}}}}}(\rho )}{\gamma }{{{{{{{{{\boldsymbol{\nabla }}}}}}}}}}\otimes {{{{{{{{{\bf{v}}}}}}}}}}\\ 	 -\frac{{k}_{{{{{{{{{{\rm{B}}}}}}}}}}}T}{{\gamma }^{2}{\varrho }_{0}m{D}_{{{{{{{{{{\rm{R}}}}}}}}}}}}{{{{{{{{{\boldsymbol{\nabla }}}}}}}}}}\otimes ({{{{{{{{{\boldsymbol{\nabla }}}}}}}}}}\cdot ({v}_{{{{{{{{{{\rm{ld}}}}}}}}}}}(\rho )\rho {{{{{{{{{\boldsymbol{\nabla }}}}}}}}}}\otimes {{{{{{{{{\bf{v}}}}}}}}}}))\\ 	+\frac{{k}_{{{{{{{{{{\rm{B}}}}}}}}}}}T{A}_{1}}{{\gamma }^{3}{\varrho }_{0}{m}^{2}{D}_{{{{{{{{{{\rm{R}}}}}}}}}}}}{{{{{{{{{\boldsymbol{\nabla }}}}}}}}}}\otimes {{{{{{{{{\boldsymbol{\nabla }}}}}}}}}}(\rho (({{{{{{{{{\bf{v}}}}}}}}}}\cdot {{{{{{{{{\boldsymbol{\nabla }}}}}}}}}})\rho ))$$

with

100 $${v}_{{{{{{{{{{\rm{D}}}}}}}}}}}[\rho,{{{{{{{{{\bf{v}}}}}}}}}}]={v}_{{{{{{{{{{\rm{ld}}}}}}}}}}}(\rho )-\frac{{A}_{4}}{\gamma m}{{{{{{{{{{\boldsymbol{\nabla }}}}}}}}}}}^{2}\rho+\frac{{A}_{1}}{{\gamma }^{2}m}({{{{{{{{{\bf{v}}}}}}}}}}\cdot {{{{{{{{{\boldsymbol{\nabla }}}}}}}}}})\rho .$$

Equation (100) provides a microscopic expression for the density-dependent swimming speed *v*_D_ in the active fluid. To see this, note that one can calculate the density-dependent swimming speed from the interaction-expansion method by looking for a contribution of the form ${{{{{{{{{\boldsymbol{\nabla }}}}}}}}}}\cdot ({v}_{{{{{{{{{{\rm{D}}}}}}}}}}}[\rho ]\hat{{{{{{{{{{\bf{u}}}}}}}}}}}\varrho )$ in the dynamic equation for *ϱ*^48^. Inserting Eq. (75) into Eq. (68) gives

101 $$\dot{\varrho }=-{{{{{{{{{\boldsymbol{\nabla }}}}}}}}}}\cdot ({{{{{{{{{\bf{v}}}}}}}}}}\varrho )-{{{{{{{{{\boldsymbol{\nabla }}}}}}}}}}\cdot (\hat{{{{{{{{{{\bf{u}}}}}}}}}}}\cdot {\underline{{{{{{{{{{\boldsymbol{v}}}}}}}}}}}}_{{{{{{{{{{\bf{P}}}}}}}}}}}\varrho )+{D}_{{{{{{{{{{\rm{R}}}}}}}}}}}{\partial }_{\varphi }^{2}\varrho .$$

This shows that the role of the density-dependent swimming speed is, in our extended theory, played by the tensorial quantity ${\underline{{{{{{{{{{\boldsymbol{v}}}}}}}}}}}}_{{{{{{{{{{\bf{P}}}}}}}}}}}$. The part of ${\underline{{{{{{{{{{\boldsymbol{v}}}}}}}}}}}}_{{{{{{{{{{\bf{P}}}}}}}}}}}$ that is proportional to ${\mathbb{1}}$ then directly gives us *v*_D_. Note that the fact that ${\underline{{{{{{{{{{\boldsymbol{v}}}}}}}}}}}}_{{{{{{{{{{\bf{P}}}}}}}}}}}$ gives rise to the density-dependent swimming speed (which is responsible for MIPS^55^) is also plausible since, as discussed in the Results, ${\underline{{{{{{{{{{\boldsymbol{v}}}}}}}}}}}}_{{{{{{{{{{\bf{P}}}}}}}}}}}$ accounts for the violation of local equilibrium. Interestingly, the density-dependent swimming speed depends not only on the density *ρ*, but also on the velocity **v**. This suggests that **v** also has to be taken into account when describing the emergence of MIPS in underdamped active fluids.

Inserting Eqs. (98)–(100) into Eq. (87) and neglecting terms of higher than second order in gradients gives Eq. (2). The reason that terms of second order in gradients are sufficient is that all third-order terms would include also **v**, which (as is evident from Eq. (3)) is of at least first order in gradients.

Deriving Eq. (3) is slightly more involved. First, we deal with the term ${D}_{{{{{{{{{{\rm{R}}}}}}}}}}}{{{{{{{{{\bf{P}}}}}}}}}}\cdot {\underline{{{{{{{{{{\boldsymbol{v}}}}}}}}}}}}_{{{{{{{{{{\bf{P}}}}}}}}}}}/(2\rho )$ appearing in Eq. (89). Inserting Eqs. (98)–(100), dropping terms of higher than third order in gradients, terms quadratic in **v** that are of higher than first order in gradients (since **v** is of first order in gradients), terms of higher than second order in fields, and products of density gradients and velocities (these approximations will be referred to as standard approximations from here on) gives

102 $$\frac{{D}_{{{{{{{{{{\rm{R}}}}}}}}}}}{{{{{{{{{\bf{P}}}}}}}}}}\cdot {\underline{{{{{{{{{{\boldsymbol{v}}}}}}}}}}}}_{{{{{{{{{{\bf{P}}}}}}}}}}}}{2\rho }=	-\frac{{v}_{0}^{2}{{{{{{{{{\boldsymbol{\nabla }}}}}}}}}}\rho }{2\rho }+\frac{3{v}_{0}{A}_{1}{{{{{{{{{\boldsymbol{\nabla }}}}}}}}}}\rho }{2\gamma m}-\frac{{A}_{1}^{2}}{2{\gamma }^{2}{m}^{2}}{{{{{{{{{\boldsymbol{\nabla }}}}}}}}}}{\rho }^{2}\\ 	+\frac{{v}_{0}}{4\rho }\,\left(\frac{2{A}_{4}}{\gamma m}-\frac{{v}_{0}{k}_{{{{{{{{{{\rm{B}}}}}}}}}}}T}{\gamma {\varrho }_{0}m{D}_{{{{{{{{{{\rm{R}}}}}}}}}}}}\right){{{{{{{{{\boldsymbol{\nabla }}}}}}}}}}{({{{{{{{{{\boldsymbol{\nabla }}}}}}}}}}\rho )}^{2}+\frac{{v}_{0}{A}_{4}}{2\gamma m\rho }({{{{{{{{{\boldsymbol{\nabla }}}}}}}}}}\rho )({{{{{{{{{{\boldsymbol{\nabla }}}}}}}}}}}^{2}\rho )\\ 	+\frac{{v}_{0}{A}_{4}}{2\gamma m\rho }\,\left({{{{{{{{{\boldsymbol{\nabla }}}}}}}}}}{({{{{{{{{{\boldsymbol{\nabla }}}}}}}}}}\rho )}^{2}+3\rho {{{{{{{{{\boldsymbol{\nabla }}}}}}}}}}{{{{{{{{{{\boldsymbol{\nabla }}}}}}}}}}}^{2}\rho+({{{{{{{{{\boldsymbol{\nabla }}}}}}}}}}\rho )({{{{{{{{{{\boldsymbol{\nabla }}}}}}}}}}}^{2}\rho )\right)\\ 	 -\frac{{v}_{0}^{2}{k}_{{{{{{{{{{\rm{B}}}}}}}}}}}T}{4\gamma {\varrho }_{0}m{D}_{{{{{{{{{{\rm{R}}}}}}}}}}}\rho }({{{{{{{{{\boldsymbol{\nabla }}}}}}}}}}{({{{{{{{{{\boldsymbol{\nabla }}}}}}}}}}\rho )}^{2}+2\rho {{{{{{{{{\boldsymbol{\nabla }}}}}}}}}}{{{{{{{{{{\boldsymbol{\nabla }}}}}}}}}}}^{2}\rho )+\frac{{v}_{{{{{{{{{{\rm{ld}}}}}}}}}}}^{2}(\rho )}{2\gamma }{{{{{{{{{{\boldsymbol{\nabla }}}}}}}}}}}^{2}{{{{{{{{{\bf{v}}}}}}}}}},$$

where we have used

103 $$({{{{{{{{{\boldsymbol{\nabla }}}}}}}}}}\otimes {{{{{{{{{\boldsymbol{\nabla }}}}}}}}}}\rho )\cdot {{{{{{{{{\boldsymbol{\nabla }}}}}}}}}}\rho=\frac{1}{2}{{{{{{{{{\boldsymbol{\nabla }}}}}}}}}}{({{{{{{{{{\boldsymbol{\nabla }}}}}}}}}}\rho )}^{2}.$$

We have not expanded the expression *v*_ld_(*ρ*)^2^ in the last term of Eq. (102) to simplify the notation even though this term thereby contains terms up to third order in fields. The first term on the right-hand side of Eq. (102) can be rewritten using $({{{{{{{{{\boldsymbol{\nabla }}}}}}}}}}\rho )/\rho={{{{{{{{{\boldsymbol{\nabla }}}}}}}}}}\ln (\rho )$. In the fourth-from-last, third-from-last, and penultimate terms, we replace *ρ* by *ϱ*_0_ in the denominator such that these terms are of second order in *ρ* as required. This yields

104 $$\frac{{{{{{{{{{\bf{P}}}}}}}}}}\cdot {\underline{{{{{{{{{{\boldsymbol{v}}}}}}}}}}}}_{{{{{{{{{{\bf{P}}}}}}}}}}}}{2\rho }=	-\frac{{v}_{0}^{2}}{2}{{{{{{{{{\boldsymbol{\nabla }}}}}}}}}}\ln (\rho )+\frac{3{v}_{0}{A}_{1}}{2\gamma m}{{{{{{{{{\boldsymbol{\nabla }}}}}}}}}}\rho -\frac{{A}_{1}^{2}}{2{\gamma }^{2}{m}^{2}}{{{{{{{{{\boldsymbol{\nabla }}}}}}}}}}{\rho }^{2}\\ 	+\,\left(\frac{3{v}_{0}{A}_{4}}{2\gamma m}-\frac{{v}_{0}^{2}{k}_{{{{{{{{{{\rm{B}}}}}}}}}}}T}{2\gamma {\varrho }_{0}m{D}_{{{{{{{{{{\rm{R}}}}}}}}}}}}\right){{{{{{{{{\boldsymbol{\nabla }}}}}}}}}}{{{{{{{{{{\boldsymbol{\nabla }}}}}}}}}}}^{2}\rho \\ 	+\,\left(\frac{{v}_{0}{A}_{4}}{\gamma m{\varrho }_{0}}-\frac{{v}_{0}^{2}{k}_{{{{{{{{{{\rm{B}}}}}}}}}}}T}{2\gamma {\varrho }_{0}^{2}m{D}_{{{{{{{{{{\rm{R}}}}}}}}}}}}\right){{{{{{{{{\boldsymbol{\nabla }}}}}}}}}}{({{{{{{{{{\boldsymbol{\nabla }}}}}}}}}}\rho )}^{2}\\ 	+\frac{{v}_{0}{A}_{4}}{\gamma m{\varrho }_{0}}({{{{{{{{{\boldsymbol{\nabla }}}}}}}}}}\rho )({{{{{{{{{{\boldsymbol{\nabla }}}}}}}}}}}^{2}\rho )+\frac{{v}_{{{{{{{{{{\rm{ld}}}}}}}}}}}^{2}(\rho )}{2\gamma }{{{{{{{{{{\boldsymbol{\nabla }}}}}}}}}}}^{2}{{{{{{{{{\bf{v}}}}}}}}}}.$$

Next, we consider the term $({\underline{{{{{{{{{{\boldsymbol{v}}}}}}}}}}}}_{{{{{{{{{{\bf{P}}}}}}}}}}}\cdot {{{{{{{{{\boldsymbol{\nabla }}}}}}}}}})\cdot {\underline{{{{{{{{{{\boldsymbol{v}}}}}}}}}}}}_{{{{{{{{{{\bf{P}}}}}}}}}}}/2$. Inserting Eqs. (4), (99) and (100) gives with the standard approximations

105 $$\frac{1}{2}({\underline{{{{{{{{{{\boldsymbol{v}}}}}}}}}}}}_{{{{{{{{{{\bf{P}}}}}}}}}}}\cdot {{{{{{{{{\boldsymbol{\nabla }}}}}}}}}})\cdot {\underline{{{{{{{{{{\boldsymbol{v}}}}}}}}}}}}_{{{{{{{{{{\bf{P}}}}}}}}}}}=	-\frac{{v}_{0}{A}_{1}}{2\gamma m}{{{{{{{{{\boldsymbol{\nabla }}}}}}}}}}\rho+\frac{{A}_{1}^{2}}{4{\gamma }^{2}{m}^{2}}{{{{{{{{{\boldsymbol{\nabla }}}}}}}}}}{\rho }^{2}\\ 	 -\,\left(\frac{3{v}_{0}{A}_{4}}{2\gamma m}-\frac{{v}_{0}^{2}{k}_{{{{{{{{{{\rm{B}}}}}}}}}}}T}{2\gamma {\varrho }_{0}m{D}_{{{{{{{{{{\rm{R}}}}}}}}}}}}\right){{{{{{{{{\boldsymbol{\nabla }}}}}}}}}}{{{{{{{{{{\boldsymbol{\nabla }}}}}}}}}}}^{2}\rho \\ 	+\,\left(\frac{3{A}_{1}{A}_{4}}{2{\gamma }^{2}{m}^{2}}-\frac{3{v}_{0}{A}_{1}{k}_{{{{{{{{{{\rm{B}}}}}}}}}}}T}{2{\gamma }^{2}{\varrho }_{0}{m}^{2}{D}_{{{{{{{{{{\rm{R}}}}}}}}}}}}\right){{{{{{{{{\boldsymbol{\nabla }}}}}}}}}}(\rho {{{{{{{{{{\boldsymbol{\nabla }}}}}}}}}}}^{2}\rho )\\ 	+\,\left(\frac{{A}_{1}{A}_{4}}{2{\gamma }^{2}{m}^{2}}-\frac{5{v}_{0}{A}_{1}{k}_{{{{{{{{{{\rm{B}}}}}}}}}}}T}{4{\gamma }^{2}{\varrho }_{0}{m}^{2}{D}_{{{{{{{{{{\rm{R}}}}}}}}}}}}\right){{{{{{{{{\boldsymbol{\nabla }}}}}}}}}}{({{{{{{{{{\boldsymbol{\nabla }}}}}}}}}}\rho )}^{2}\\ 	 -\,\,\left(\frac{{A}_{1}{A}_{4}}{{\gamma }^{2}{m}^{2}}-\frac{{v}_{0}{A}_{1}{k}_{{{{{{{{{{\rm{B}}}}}}}}}}}T}{2{\gamma }^{2}{\varrho }_{0}{m}^{2}{D}_{{{{{{{{{{\rm{R}}}}}}}}}}}}\right)({{{{{{{{{\boldsymbol{\nabla }}}}}}}}}}\rho )({{{{{{{{{{\boldsymbol{\nabla }}}}}}}}}}}^{2}\rho )\\ 	 -\frac{{v}_{{{{{{{{{{\rm{ld}}}}}}}}}}}{(\rho )}^{2}}{2\gamma }{{{{{{{{{{\boldsymbol{\nabla }}}}}}}}}}}^{2}{{{{{{{{{\bf{v}}}}}}}}}},$$

where we used Eq. (103) and

106 $${{{{{{{{{{\boldsymbol{\nabla }}}}}}}}}}}^{2}{\rho }^{2}=2(\rho {{{{{{{{{{\boldsymbol{\nabla }}}}}}}}}}}^{2}\rho+{({{{{{{{{{\boldsymbol{\nabla }}}}}}}}}}\rho )}^{2}),$$

107 $$\rho {{{{{{{{{\boldsymbol{\nabla }}}}}}}}}}{{{{{{{{{{\boldsymbol{\nabla }}}}}}}}}}}^{2}\rho={{{{{{{{{\boldsymbol{\nabla }}}}}}}}}}(\rho {{{{{{{{{{\boldsymbol{\nabla }}}}}}}}}}}^{2}\rho )-({{{{{{{{{\boldsymbol{\nabla }}}}}}}}}}\rho )({{{{{{{{{{\boldsymbol{\nabla }}}}}}}}}}}^{2}\rho ).$$

Finally, using Eqs. (98) and (106) and the standard approximations, we find

108 $${A}_{3}{{{{{{{{{{\boldsymbol{\nabla }}}}}}}}}}}^{2}{{{{{{{{{\bf{P}}}}}}}}}}+2{A}_{3}{{{{{{{{{\boldsymbol{\nabla }}}}}}}}}}({{{{{{{{{\boldsymbol{\nabla }}}}}}}}}}\cdot {{{{{{{{{\bf{P}}}}}}}}}})=-\frac{3{v}_{0}{A}_{3}}{{D}_{{{{{{{{{{\rm{R}}}}}}}}}}}}{{{{{{{{{\boldsymbol{\nabla }}}}}}}}}}{{{{{{{{{{\boldsymbol{\nabla }}}}}}}}}}}^{2}\rho+\frac{6{A}_{1}{A}_{3}}{\gamma m{D}_{{{{{{{{{{\rm{R}}}}}}}}}}}}{{{{{{{{{\boldsymbol{\nabla }}}}}}}}}}(\rho {{{{{{{{{{\boldsymbol{\nabla }}}}}}}}}}}^{2}\rho+{({{{{{{{{{\boldsymbol{\nabla }}}}}}}}}}\rho )}^{2}).$$

Inserting Eqs. (104), (105), and (108) into Eq. (89) and collecting terms results in

109 $$\dot{{{{{{{{{{\bf{v}}}}}}}}}}}=	-({{{{{{{{{\bf{v}}}}}}}}}}\cdot {{{{{{{{{\boldsymbol{\nabla }}}}}}}}}}){{{{{{{{{\bf{v}}}}}}}}}}-\gamma {{{{{{{{{\bf{v}}}}}}}}}}\\ 	 -\frac{1}{m}{{{{{{{{{\boldsymbol{\nabla }}}}}}}}}}\,\left(\left({k}_{{{{{{{{{{\rm{B}}}}}}}}}}}T+\frac{m{v}_{0}^{2}}{2}\right)\ln (\rho )\right.\\ 	+\,\left({A}_{2}-\frac{2{v}_{0}{A}_{1}}{\gamma }\right)\rho+\frac{3{A}_{1}^{2}}{4{\gamma }^{2}m}{\rho }^{2}\\ 	 -\,\left(\frac{3{v}_{0}{A}_{3}}{{D}_{{{{{{{{{{\rm{R}}}}}}}}}}}}+\frac{3{v}_{0}{A}_{4}}{\gamma }-\frac{{v}_{0}^{2}{k}_{{{{{{{{{{\rm{B}}}}}}}}}}}T}{\gamma {\varrho }_{0}{D}_{{{{{{{{{{\rm{R}}}}}}}}}}}}\right){{{{{{{{{{\boldsymbol{\nabla }}}}}}}}}}}^{2}\rho \\ 	 -\,\left(-\frac{6{A}_{1}{A}_{3}}{\gamma m{D}_{{{{{{{{{{\rm{R}}}}}}}}}}}}-\frac{3{A}_{1}{A}_{4}}{2{\gamma }^{2}m}+\frac{3{v}_{0}{A}_{1}{k}_{{{{{{{{{{\rm{B}}}}}}}}}}}T}{2{\gamma }^{2}{\varrho }_{0}m{D}_{{{{{{{{{{\rm{R}}}}}}}}}}}}\right)(\rho {{{{{{{{{{\boldsymbol{\nabla }}}}}}}}}}}^{2}\rho )\\ 	+\,\left(-\frac{{v}_{0}{A}_{4}}{\gamma {\varrho }_{0}}+\frac{{v}_{0}^{2}{k}_{{{{{{{{{{\rm{B}}}}}}}}}}}T}{2\gamma {\varrho }_{0}^{2}{D}_{{{{{{{{{{\rm{R}}}}}}}}}}}}+\frac{{A}_{1}{A}_{4}}{2{\gamma }^{2}m}\right.\\ 	 -\,\left.\,\left.\frac{5{v}_{0}{A}_{1}{k}_{{{{{{{{{{\rm{B}}}}}}}}}}}T}{4{\gamma }^{2}{\varrho }_{0}m{D}_{{{{{{{{{{\rm{R}}}}}}}}}}}}+\frac{6{A}_{1}{A}_{3}}{\gamma m{D}_{{{{{{{{{{\rm{R}}}}}}}}}}}}\right){({{{{{{{{{\boldsymbol{\nabla }}}}}}}}}}\rho )}^{2}+{U}_{1}\right)\\ 	+\,\left(\frac{{A}_{1}{A}_{4}}{{\gamma }^{2}{m}^{2}}-\frac{{v}_{0}{A}_{1}{k}_{{{{{{{{{{\rm{B}}}}}}}}}}}T}{2{\gamma }^{2}{\varrho }_{0}{m}^{2}{D}_{{{{{{{{{{\rm{R}}}}}}}}}}}}+\frac{{v}_{0}{A}_{4}}{\gamma m{\varrho }_{0}}\right)({{{{{{{{{\boldsymbol{\nabla }}}}}}}}}}\rho )({{{{{{{{{{\boldsymbol{\nabla }}}}}}}}}}}^{2}\rho )\\ 	+\frac{{v}_{{{{{{{{{{\rm{ld}}}}}}}}}}}{(\rho )}^{2}}{\gamma }{{{{{{{{{{\boldsymbol{\nabla }}}}}}}}}}}^{2}{{{{{{{{{\bf{v}}}}}}}}}}.$$

We have not dropped the higher-order contributions in *ρ* for the logarithmic term, which is consistent with the fact that we do not make a constant-mobility approximation^97^. It is interesting that, instead of the thermal energy *k*_B_*T* we would have in the passive case, the ideal gas contribution ${f}^{{\prime} }$ is proportional to ${k}_{{{{{{{{{{\rm{B}}}}}}}}}}}T+m{v}_{0}^{2}/2$, implying that the active contribution to the kinetic energy effectively shifts the temperature by $m{v}_{0}^{2}/(2{k}_{{{{{{{{{{\rm{B}}}}}}}}}}})$. This is a different sort of effective temperature than the one reported for active systems by Preisler and Dijkstra^57^. The additional term $m{v}_{0}^{2}\ln (\rho )/2$ originates from Eq. (104). Equation (109) can be written in the form

110 $$\dot{{{{{{{{{{\bf{v}}}}}}}}}}}+({{{{{{{{{\bf{v}}}}}}}}}}\cdot {{{{{{{{{\boldsymbol{\nabla }}}}}}}}}}){{{{{{{{{\bf{v}}}}}}}}}}=	-\frac{1}{m}{{{{{{{{{\boldsymbol{\nabla }}}}}}}}}}\,\left({f}^{{\prime} }(\rho )-({\kappa }_{0}+\varsigma \rho ){{{{{{{{{{\boldsymbol{\nabla }}}}}}}}}}}^{2}\rho -\gamma {{{{{{{{{\bf{v}}}}}}}}}}\right.\\ 	 \, \left.+{\lambda }_{0}{({{{{{{{{{\boldsymbol{\nabla }}}}}}}}}}\rho )}^{2}+{U}_{1}\right)\\ 	+\frac{{v}_{{{{{{{{{{\rm{ld}}}}}}}}}}}{(\rho )}^{2}}{\gamma }{{{{{{{{{{\boldsymbol{\nabla }}}}}}}}}}}^{2}{{{{{{{{{\bf{v}}}}}}}}}}+\frac{\xi }{m}({{{{{{{{{{\boldsymbol{\nabla }}}}}}}}}}}^{2}\rho ){{{{{{{{{\boldsymbol{\nabla }}}}}}}}}}\rho$$

with the function

111 $${f}^{{\prime} }(\rho )=\,\left({k}_{{{{{{{{{{\rm{B}}}}}}}}}}}T+\frac{m{v}_{0}^{2}}{2}\right)\ln (\rho )+\,\left({A}_{2}-\frac{2{v}_{0}{A}_{1}}{\gamma }\right)\rho+\,\left(\frac{3{A}_{1}^{2}}{4{\gamma }^{2}m}\right){\rho }^{2}$$

and the coefficients

112 $${\kappa }_{0}=\frac{3{v}_{0}{A}_{3}}{{D}_{{{{{{{{{{\rm{R}}}}}}}}}}}}+\frac{3{v}_{0}{A}_{4}}{\gamma }-\frac{{v}_{0}^{2}{k}_{{{{{{{{{{\rm{B}}}}}}}}}}}T}{\gamma {\varrho }_{0}{D}_{{{{{{{{{{\rm{R}}}}}}}}}}}},$$

113 $$\varsigma=-\frac{6{A}_{1}{A}_{3}}{\gamma m{D}_{{{{{{{{{{\rm{R}}}}}}}}}}}}-\frac{3{A}_{1}{A}_{4}}{2{\gamma }^{2}m}+\frac{3{v}_{0}{A}_{1}{k}_{{{{{{{{{{\rm{B}}}}}}}}}}}T}{2{\gamma }^{2}{\varrho }_{0}m{D}_{{{{{{{{{{\rm{R}}}}}}}}}}}},$$

114 $${\lambda }_{0}=\, -\frac{{v}_{0}{A}_{4}}{\gamma {\varrho }_{0}}+\frac{{v}_{0}^{2}{k}_{{{{{{{{{{\rm{B}}}}}}}}}}}T}{2\gamma {\varrho }_{0}^{2}{D}_{{{{{{{{{{\rm{R}}}}}}}}}}}}+\frac{{A}_{1}{A}_{4}}{2{\gamma }^{2}m}\\ -\frac{5{v}_{0}{A}_{1}{k}_{{{{{{{{{{\rm{B}}}}}}}}}}}T}{4{\gamma }^{2}{\varrho }_{0}m{D}_{{{{{{{{{{\rm{R}}}}}}}}}}}}+\frac{6{A}_{1}{A}_{3}}{\gamma m{D}_{{{{{{{{{{\rm{R}}}}}}}}}}}},$$

115 $$\xi=\frac{{A}_{1}{A}_{4}}{{\gamma }^{2}m}-\frac{{v}_{0}{A}_{1}{k}_{{{{{{{{{{\rm{B}}}}}}}}}}}T}{2{\gamma }^{2}{\varrho }_{0}m{D}_{{{{{{{{{{\rm{R}}}}}}}}}}}}+\frac{{v}_{0}{A}_{4}}{\gamma {\varrho }_{0}}.$$

Finally, we separate the variational and non-variational dynamics using an argument adapted from Wittkowski et al.^14^. While −*ρ***∇**^2^*ρ* is non-variational, one could write − (*ρ***∇**^2^*ρ* + (**∇***ρ*)^2^/2) as a derivative of the free energy density *ρ*(**∇***ρ*)^2^/2. On this basis, we replace − *ςρ***∇**^2^*ρ* by − *ςρ***∇**^2^*ρ* − *ς*(**∇***ρ*)^2^/2 + *ς*(**∇***ρ*)^2^/2 and combine the last term (i.e., *ς*(**∇***ρ*)^2^/2) with the term *λ*_0_(**∇***ρ*)^2^ already present to get a term (*λ*_0_ + *ς*/2)(**∇***ρ*)^2^. The remaining gradient contribution − (*κ*_0_ + *ςρ*)**∇**^2^*ρ* − *ς*(**∇***ρ*)^2^/2 can be written as a functional derivative of the passive free energy *F*_*P*_ = ∫ d^2^*rκ*(*ρ*)(**∇***ρ*)^2^/2 with *κ*(*ρ*) = *κ*_0_ + *ςρ*. As is standard in passive model B^14^, we make the simplifying assumption that *κ* is constant. Also, we define *λ*_0_ + *ς*/2 = *λ*. Then, Eq. (110) reduces to Eq. (3) of AMI+. Note that, for large values of ${v}_{0}^{2}{k}_{{{{{{{{{{\rm{B}}}}}}}}}}}T/{\varrho }_{0}$, Eq. (20) used in the derivation of Eq. (29) follows from Eqs. (112) and (114) together with *ρ*_0_ = 2*ϱ*_0_ (see Eq. (19)). Hence, the choice of coefficients used in the derivation of an analog of the Schrödinger equation is quite natural for large activities or temperatures.

Having available a microscopic theory, we can now understand in more detail the significance of the limit *γ* → 0 that has been used above to derive an analog of the Schrödinger equation. Simply setting *γ* = 0 is problematic for two reasons: First, the microscopic model given by Eqs. (55)–(61) reduces to a passive system for *γ* = 0, which appears to be in conflict with the fact that Eq. (18), whose derivation requires (among other things) setting *γ* = 0 in Eq. (9), still contains the active term *λ*(**∇***ρ*)^2^. Second, the microscopic definitions of ${f}^{{\prime} }$ and the model coefficients given by Eqs. (111)–(115) have *γ* in the denominator in several terms.

To understand this issue, note first that in a passive system (which we have if *γ* is exactly zero), **P** and ${\underline{{{{{{{{{{\boldsymbol{v}}}}}}}}}}}}_{{{{{{{{{{\bf{P}}}}}}}}}}}$ cannot influence the dynamics since we have spherical particles. For determining the form of Eq. (9) in the case *γ* = 0, the best strategy is thus to re-do the coarse graining procedure with **P** = **0** and ${\underline{{{{{{{{{{\boldsymbol{v}}}}}}}}}}}}_{{{{{{{{{{\bf{P}}}}}}}}}}}=\underline{{{{{{{{{{\boldsymbol{0}}}}}}}}}}}$, giving us

116 $$\dot{{{\bf{v}}}}+({{{{{{{{{\bf{v}}}}}}}}}}\cdot {{{{{{{{{\boldsymbol{\nabla }}}}}}}}}}){{{{{{{{{\bf{v}}}}}}}}}}=-\frac{1}{m}{{{{{{{{{\boldsymbol{\nabla }}}}}}}}}}({\;f}^{{\prime} }(\rho )+{U}_{1}).$$

(This does not mean that there can be no gradient terms in the passive case. These can be obtained by a more sophisticated treatment of interaction terms).

Of course, the correct passive limit should also emerge from the full theory (assuming that all approximations made during the derivation remain valid in this limit). Whether this is the case is not obvious since the coefficients *A*_1_, …, *A*_4_, which we have treated as constants, generally vary if quantities like *γ* or *v*_0_ change. While the precise dependencies remain to be investigated, we may assume that *A*_1_, *A*_3_, and *A*_4_ become small in a passive system due to the resulting approximate rotational symmetry of *g* (see Eqs. (83), (85), and (86)). Taking this into account, it is easy to see from Eqs. (111)–(115) that Eq. (3) (and thus also Eq. (9)) indeed reduces to Eq. (116) (with an additional term − *γ***v**) for *v*_0_ → 0.

The case *γ* → 0 is a bit more difficult since the microscopic derivation of AMI+ involves divisions by *γ*. This is the reason for why *γ* appears in the denominator in several terms of Eqs. (111)–(115), and this also implies that we cannot simply set *γ* = 0 since this would require a division by zero. Nevertheless, we can recover Eq. (116) if we first take the limit *v*_0_ → 0 and then *γ* → 0. (Note that the blow-up apparently occurring in Eqs. (111)–(115) in the limit *γ* → 0 will be compensated for by the small values of *A*_1_, *A*_3_, and *A*_4_, and by the fact that the assumption that the polarization relaxes very quickly implies a large value of *D*_R_).

Here, however, we wish to study the effects of activity and are therefore not interested in the limit (116). Instead, we consider a small but finite *γ*, and at the same time—to ensure that the terms proportional *κ* and *λ* do not become negligible compared to ${f}^{{\prime} }$—assume that *v*_0_ is very large. In this case, we may assume (as done in Eq. (18)) that the (partly) passive term involving ${f}^{{\prime} }$ can be neglected compared to the active terms. Consequently, the limit *γ* → 0 that has been investigated in the Results is not the rather trivial frictionless (passive) limit, but the case of small but finite damping and strong activity. Interestingly, our investigation of the active tunnel effect has solely relied on the form (11) of *μ*, which (as remarked above) also occurs in the overdamped AMB. Consequently, this effect may occur both in weakly damped and in overdamped active matter.

### Nondimensionalization

Here, we derive the nondimensional static equations required for our analysis of the tunnel effect, starting with the quantum model. Using the Madelung transformations, Eq. (37) can be rewritten as^88^

117 $$0={\partial }_{x}({\rho }_{{{{{{{{{{\rm{q}}}}}}}}}}}v),$$

118 $$E=\frac{m}{2}{v}^{2}-\frac{{\hslash }^{2}}{2m}\,\left(\frac{1}{2}{\partial }_{x}^{2}\ln ({\rho }_{{{{{{{{{{\rm{q}}}}}}}}}}})+\frac{1}{4}{({\partial }_{x}\ln ({\rho }_{{{{{{{{{{\rm{q}}}}}}}}}}}))}^{2}\right)+{U}_{1}.$$

For *v* = 0, Eq. (118) simplifies to

119 $$E=-\frac{{\hslash }^{2}}{2m}\,\left(\frac{1}{2}{\partial }_{x}^{2}\ln ({\rho }_{{{{{{{{{{\rm{q}}}}}}}}}}})+\frac{1}{4}{({\partial }_{x}\ln ({\rho }_{{{{{{{{{{\rm{q}}}}}}}}}}}))}^{2}\right)+{U}_{1}.$$

While *v* is not zero for the quantum-mechanical tunnel effect^88^, the essential physics can still be captured in the simpler case *v* = 0. Defining $E={E}_{0}\tilde{E}$, ${U}_{1}={E}_{0}{\tilde{U}}_{1}$, and $x={x}_{0}\tilde{x}$ (where the tilde denotes dimensionless quantities and *E*_0_ and *x*_0_ are constants) gives

120 $$\tilde{E}=-\frac{{\tilde{\hslash }}^{2}}{2\tilde{m}}\,\left(\frac{1}{2}{\partial }_{\tilde{x}}^{2}\ln ({\rho }_{{{{{{{{{{\rm{q}}}}}}}}}}})+\frac{1}{4}{({\partial }_{\tilde{x}}\ln ({\rho }_{{{{{{{{{{\rm{q}}}}}}}}}}}))}^{2}\right)+{\tilde{U}}_{1}$$

with ${\tilde{\hslash }}^{2}/(2\tilde{m})={\hslash }^{2}/(2m{E}_{0}{x}_{0}^{2})$. Dropping all tildes results in Eq. (44).

Note that we have not nondimensionalized the density *ρ*_q_ in the argument of the logarithm since, strictly speaking, it is already nondimensionalized (as noted above, $\ln ({\rho }_{{{{{{{{{{\rm{q}}}}}}}}}}})$ is a short notation for $\ln ({\rho }_{{{{{{{{{{\rm{q}}}}}}}}}}}/{\rho }_{0})$). Moreover, the wavefunctions and densities given in Eqs. (39), (42), and (43) are dimensionless (this is standard in treatments of the tunnel effect and a consequence of the fact that plane wave solutions of Eq. (37) cannot be normalized on infinite domains).

Next, we turn to the static active matter model (11). Defining $\rho={\rho }_{0}\tilde{\rho }$, $\mu={E}_{0}\tilde{\mu }$, ${f}^{{\prime} }={E}_{0}{\tilde{\,f}}^{{\prime} }$, ${U}_{1}={E}_{0}{\tilde{U}}_{1}$, and $x={x}_{0}\tilde{x}$ (the tildes again denote dimensionless quantities and *ρ*_0_ is another constant) gives

121 $$\tilde{\mu }={\tilde{f}}^{{\prime} }(\tilde{\rho })-\tilde{\kappa }{\partial }_{\tilde{x}}^{2}\tilde{\rho }+\tilde{\lambda }{({\partial }_{\tilde{x}}\tilde{\rho })}^{2}+{\tilde{U}}_{1}$$

with $\tilde{\kappa }={\kappa {\rho }_{0}}/({{x}_{0}}^{2}{E}_{0})$ and $\tilde{\lambda }={{\lambda {\rho }_{0}}^{2}}/({{x}_{0}}^{2}{E}_{0})$. Dropping all tildes results in Eq. (45). We could have eliminated the parameters *κ* and *λ* (corresponding to *ℏ*^2^/(2*m*) in Eq. (44)) by an appropriate choice of the constants *ρ*_0_, *E*_0_, and *x*_0_, but we assume here that these constants have already been used to eliminate other parameters, e.g., in the free energy.

### Numerical path continuation

To obtain the results shown in Fig. 2, we use numerical path continuation via the Matlab package pde2path^98^. Starting from the analytical solution (Eq. (47)) of model (53), pde2path subsequently applies tangent predictors and Newton correctors to track a branch of steady states through parameter space. A numerical linear stability analysis during the continuation yields the stability of the corresponding solution and enables the detection of bifurcations. Pde2path uses the finite element method and the model is implemented in a weak formulation. We have used a primary control parameter (*a*, *κ*, or *λ*) and the chemical potential *μ* as a secondary one which is adapted freely during the continuation to ensure mass conservation.

## Data Availability

The data corresponding to Fig. 2 generated in this study have been deposited in the Zenodo database under accession code 10.5281/zenodo.6376060^99^.

## Code availability

The code used for the numerical continuation has been deposited in the Zenodo database under accession code 10.5281/zenodo.6376060^99^.
